# Untargeted Profiling of Bile Acids and Lysophospholipids Identifies the Lipid Signature Associated with Glycemic Outcome in an Obese Non-Diabetic Clinical Cohort

**DOI:** 10.3390/biom10071049

**Published:** 2020-07-15

**Authors:** Nicolas Christinat, Armand Valsesia, Mojgan Masoodi

**Affiliations:** 1 Nestlé Research, Nestlé Institute of Health Sciences, 1015 Lausanne, Switzerland; Nicolas.Christinat@rd.nestle.com (N.C.); Armand.valsesia@gmail.com (A.V.); 2 Institute of Clinical Chemistry, Inselspital, Bern University Hospital, 3010 Bern, Switzerland

**Keywords:** lipidomics, liquid chromatography, mass spectrometry, bile acids, lysophospholipids, weight loss, glycemic control

## Abstract

The development of high throughput assays for assessing lipid metabolism in metabolic disorders, especially in diabetes research, nonalcoholic fatty liver disease (NAFLD), and nonalcoholic steatohepatitis (NASH), provides a reliable tool for identifying and characterizing potential biomarkers in human plasma for early diagnosis or prognosis of the disease and/or responses to a specific treatment. Predicting the outcome of weight loss or weight management programs is a challenging yet important aspect of such a program’s success. The characterization of potential biomarkers of metabolic disorders, such as lysophospholipids and bile acids, in large human clinical cohorts could provide a useful tool for successful predictions. In this study, we validated an LC-MS method combining the targeted and untargeted detection of these lipid species. Its potential for biomarker discovery was demonstrated in a well-characterized overweight/obese cohort subjected to a low-caloric diet intervention, followed by a weight maintenance phase. Relevant markers predicting successful responses to the low-caloric diet intervention for both weight loss and glycemic control improvements were identified. The response to a controlled weight loss intervention could be best predicted using the baseline concentration of three lysophospholipids (PC(22:4/0:0), PE(17:1/0:0), and PC(22:5/0:0)). Insulin resistance on the other hand could be best predicted using clinical parameters and levels of circulating lysophospholipids and bile acids. Our approach provides a robust tool not only for research purposes, but also for clinical practice, as well as designing new clinical interventions or assessing responses to specific treatment. Considering this, it presents a step toward personalized medicine.

## 1. Introduction

Lipidomics has become an increasingly important tool in metabolic disorders and diabetes research for the detection of biomarkers in human plasma for early diagnosis or prognosis of the prediabetic state. Lysophosphospholipids (LPL) have been detected as potential markers for identifying prediabetic patients. In particular, an association between lower levels of circulating LPC(16:0), LPC(16:1), LPC(18:2), and LPC(20:0) and obesity, insulin resistance, and type 2 diabetes has been reported [1,2,3]. These lipid species are mainly formed from phospholipids upon the activation of phospholipases A1 and A2. They can act as signaling lipids and exhibit a wide range of biological effects. In particular, their roles in the regulation of inflammation [4,5] and activation of the transcription factor peroxisome proliferator-activated receptor (PPAR)δ have been reported [6].

Bile acids (BAs) are also recognized as key regulators of metabolism and can be used as potential biomarkers in metabolic disorders [7,8]. Bile acids are synthetized in the liver from cholesterol via the cytochrome 7α hydroxylase (Cyp7A) pathway and a mitochondrial cytochrome 27α hydroxylase (Cyp27A), and then conjugated with taurine or glycine in the liver, forming bile salts, which are secreted into the bile. They assist with lipid digestion and absorption once released into the intestine [9]. In addition, BAs regulate glucose homeostasis by acting directly on farnesoid X receptor (FXR) and Takeda G protein-coupled receptor 5 (TGR5) in the intestine and liver, as well as regulating the production and secretion of GLP-1 via opposite effects on TGR5 and FXR [10,11,12]. Due to the importance of these lipid species in the regulation of lipid and glucose metabolism, it is very important to develop robust assays for identifying and detecting alterations in their levels in order to assess their impacts on human metabolism.

Bile acids have traditionally been analyzed by reversed phase chromatography coupled to tandem mass spectrometry [13]. This methodology allows for the separation of multiple bile acid isomers and usually covers primary, secondary, and glycine and taurine conjugated bile acids in matrices such as serum [14], bile [15], plasma, and urine [16]. Because unconjugated bile acids do not fragment well, their analysis by LC-MS/MS is often performed without fragmentation, greatly reducing the specificity of this approach. Another drawback of quantification by tandem mass spectrometry is the limited number of targeted analytes. In recent years, high-resolution mass spectrometry (HRMS) has been increasingly used for bile acid profiling and quantification [17,18]. Using this technique, the number of profiled bile acids has dramatically increased: Sulfated and glucuronidated bile acids are now commonly profiled, allowing for a better understanding of bile acid metabolism [19,20,21].

The simultaneous profiling of bile acids and lysophospholipids can be an extremely powerful tool for studying lipid metabolism and diseases associated with it. Lipidomics methods can achieve a broad coverage of lipid and bile acid species, but molecular species may not be fully characterized [22]. In addition, long gradients are often required to achieve a satisfactory separation of lipid isomers, which complicates their application to large clinical cohorts [23,24,25].

The method described herein combines the detection of bile acids and lysophospholipids in a quantitative and non-targeted manner. The method allows for the separation of bile acids, as well as lysophospholipid isomers. In particular, LPL positional isomers are well-separated, allowing for the profiling of about 90 LPL and 40 bile acids in human plasma. Its relatively short runtime (<20 min) makes it well-suited for capturing changes in the lipid profile associated with weight loss or weight management programs in large human clinical cohorts. We applied this method to well-characterized overweight/obese subjects who were subjected to an 8-week low-caloric diet (LCD) intervention, followed by a 6-month weight maintenance phase (DiOGenes study) [26], to evaluate the feasibility of identifying potential markers predicting glycemic non-responders.

## 2. Materials and Methods

### 2.1. Chemicals and Reagents

Methanol was purchased from Merck (Darmstadt, Germany) and water was purified in-house using a Milli-Q Advantage A10 system from Merck Millipore (Billerica, MA, USA). Ammonium acetate and acetic acids were supplied by Sigma-Aldrich (St. Louis, MO, USA). Bile acid standards were purchased from Sigma-Aldrich (St. Louis, MO, USA), Toronto Research Chemicals (North York, Canada), Cayman Chemicals (Ann Arbor, MI, USA), and Steraloids (Newport, RI, USA). Lysophospholipid standards were purchased from Avanti Polar Lipids (Alabaster, AL, USA). Refer to Tables S1 and S7 for details about standards.

### 2.2. Preparation of Standard Solutions

Individual internal standard stock solutions were prepared from commercially available material. The stock solutions were then combined to prepare two internal standard mixtures in methanol: One for bile acids and one for lysophospholipids. The bile acid internal standard mixture contained the following species at a concentration of 25 µM: CA-d_5_, CDCA-d_4_, DCA-d_5_, LCA-d_4_, UDCA-d_4_, HDCA-d_5_, GCA-d_5_, GCDCA-d_7_, GDCA-d_4_, GUDCA-d_5_, TCA-d_5_, TDCA-d_5_, TLCA-d_5_, TCDCA-d_5_, and TUDCA-d_5_. The lysophospholipid internal standard mixture contained PC (13:0/0:0), PC (18:1-d7/0:0), PE (13:0/0:0), PE (18:1-d7/0:0), PI (13:0/0:0), PI (17:1/0:0), PS (13:0/0:0), PS (17:1/0:0), PG (13:0/0:0), and PG (17:1/0:0) at concentrations ranging from 0.1 to 1.2 µM.

A mixture of external standards in methanol (250 µM) was prepared from individual stock solutions. The mixture was used to prepared calibration standards in solvent (0.003–5 µM), as well as QC samples.

QC samples (0.002, 0.2, and 2 µM) were prepared by spiking charcoal stripped plasma with different volumes of external standard solution.

### 2.3. Lipid Extraction

During all preparation steps, instruments were maintained at 4 °C and samples were stored on ice to prevent lipid degradation. For the same reason, frozen plasma samples were thawed at 4 °C. Once thawed, samples were briefly shaken before 50 µL was transferred in a 1.5 mL snap-lock tube. Proteins were precipitated by adding 500 µL methanol (with pre-mixed internal standards). The suspension was mixed for 5 min at 100 rpm with a Thermomixer C and centrifuged for 15 min at 17,000× *g*. A total of 440 µL of supernatant was transferred to a second snap-lock tube and the solvent was evaporated under vacuum. The extract was reconstituted in 40 µL of a 1:1 mixture of eluents A and B, transferred in a vial equipped with a low volume insert, and immediately injected.

### 2.4. Liquid Chromatography Separation and Mass Spectrometry Detection

Liquid chromatography was performed on a I-Class UPLC system (Waters Corporation, Milford, MA, USA) combining a binary pump, an FTN autosampler, and a column oven. Chromatographic separation was achieved on a Waters ACQUITY UPLC BEH Shield RP18 Column (100 × 2.1 mm, 1.7 µm) equipped with an in-line filter at a flow rate of 450 µL/min. Mobile phase A and B were 10 mM ammonium acetate + 0.01% acetic acid solutions in water and methanol, respectively. The binary solvent gradient was the following: 0.0–2.0 min at 50% B, 2.0–13.0 min from 50% to 95% B, 13.0–16.0 min 95% B, and 16.0–19.0 min 50%. The column temperature was set to 40 °C and 2 µL of sample was injected into the system.

High-resolution mass spectrometric analysis was performed on a Thermo Scientific Q Exactive Plus instrument (ThermoFisher Scientific, Bremen, Germany). The instrument was operated in negative ionization mode and detection was performed in both MS^1^ and MS^2^ (without precursor isolation) in one duty cycle (Full MS-All ions fragmentation (AIF) experiment). The instrument parameters were as follows: for MS^1^, mass range *m*/*z* 370–700, resolving power of 70,000 (at *m*/*z* = 200), automatic gain control (AGC) target 5e^6^, and maximum injection time of 120 ms, and for MS^2^, resolving power of 17,500 (at *m*/*z* = 200), AGC target 5e^6^, maximum injection time of 75 ms, and normalized collision energy (NCE) of 50.

Electrospray ionization was performed using a HESI-II probe. Source parameters were as follows: Spray voltage of −4.0 kV, heater and capillary temperatures of 350 °C, sheath gas flow rate of 45 arbitrary units (AU), auxiliary gas of 15 AU, and sweep gas of 0 AU. The instrument was calibrated according to manufacturer specifications.

### 2.5. Data Analysis

Xcalibur software 4.0 (ThermoFisher Scientific, Bremen, Germany) was used for data processing. MS^1^ chromatograms were extracted using a mass tolerance of 5 ppm and signals were integrated with the ISIS algorithm. Calibration curves were linearly fitted with a weighting factor of 1/x. An in-house developed R-based package (version 3.4.4) was used for further statistical analyses.

### 2.6. Development and Validation of the LC-MS Method

The method was developed using a set of 29 bile acid standards (Table S1) and 42 LPL standards (Table S7). Chromatographic separation and mass spectrometric detection were optimized to ensure retention time stability and detection selectivity.

The method was then validated according to FDA guidelines [27]. In particular, the following parameters were assessed: Selectivity, recovery of extraction, dynamic range, limits of detection and quantification, matrix effect, trueness, precision, and repeatability.

#### 2.6.1. Selectivity

The identity of each analyte (selectivity) was confirmed using the following criteria:

1. A signal is visible on an MS^1^ chromatogram extracted using the analyte mass and a mass tolerance of ±5 ppm;
2. A signal is visible on an MS^2^ chromatogram extracted using a characteristic fragment mass (fatty acid, phospholipid head group for lysophospholipids, and conjugated group for bile acids) and a mass tolerance of ±5 ppm;
3. The retention time of the analyte matches the result obtained with a reference standard.

#### 2.6.2. Recovery of Extraction

The recovery of extraction for each analyte was determined in triplicate at two concentrations using charcoal stripped plasma samples. Recovery was calculated by dividing the signal area in plasma spiked before extraction by the average area obtained in plasma spiked after extraction. The result is expressed as a percentage.

#### 2.6.3. Dynamic Range, Limits of Detection, and Quantification

The dynamic range and limit of quantification (LOQ) of the method were evaluated using an 8-point calibration curve for each analyte. Each calibration level was injected in triplicate. Calibration curves were constructed by calculating the peak area ratio of the analyte and IS for each analyte and concentration level and performing linear regression. A coefficient of determination (R^2^) > 0.995 calculated over a minimum of six calibration points, as well as a ±15% deviation from the nominal value (±20% for the lower limit of quantitation (LLOQ)), were considered acceptable. LOD was calculated as the lowest concentration result at a signal-to-noise ratio of 3 and LOQ was defined as the lowest concentration that could be confidently quantified (CV ≤ 20% in triplicate samples).

#### 2.6.4. Matrix Effect

The matrix effect was evaluated by building calibration curves in extracted charcoal stripped plasma and HPLC solvent. Each calibration solution was injected three times and the matrix effect was calculated by comparing the slopes of both calibration curves.

#### 2.6.5. Trueness, Precision, and Repeatability

To evaluate the trueness and precision (repeatability and intermediate precision), QC samples were analyzed in triplicate at three spiking levels. The same procedure was repeated on six different days to assess the reproducibility of the method.

### 2.7. Untargeted Screening of Bile Acids and Lysophospholipids

Bile acids and lysophospholipids were screened based on their exact mass (−H^+^ or + CH_3_CCO^−^ adducts) and fragmentation patterns and the identity of some lysophospholipids was confirmed with standards. For others, prediction of the retention time was used for identification. Finally, method parameters related to LPL detection were assessed using standard solutions and plasma samples.

### 2.8. Experimental Model and Subjects’ Details

DiOGenes is a multicenter, randomized controlled dietary intervention study involving eight European countries (ClinicalTrials.gov number, NCT00390637). The study has previously been described in detail [28]. Briefly, 938 overweight/obese, non-diabetic adults (BMI between 27 and 45 kg/m^2^, blood fasting glucose below 6.1 mmol/L) underwent an 8-week weight-loss phase using a complete meal replacement low-calorie diet (LCD). The LCD provided 800 kcal/day (Modifast, Nutrition et Santé France). Among the 781 participants who completed the LCD, 773 achieved >8% weight loss and were randomized to a 26-week weight-maintenance diet. A total of 548 subjects completed the weight-maintenance diet (WMD), among which 383 (70%) had available EDTA plasma samples at all intervention time-points: Baseline (Clinical Intervention Day 1, CID1), after 8 weeks of LCD (CID2), and after 6 months of weight maintenance (CID3). We selected 100 subjects from identified responders and non-responder groups that were previously identified [28,29]. Table 1 shows the baseline characteristics of the selected patients. Local ethics committees approved the study (Protocol CER-VD 282/14 from the commission cantonale d’ethique de la recherche sur l’etre humain. av. de Chailly 23, 1012 Lausanne), each patient provided written informed consent, and the study was carried out in accordance with the principles of the Declaration of Helsinki.

### 2.9. Statistical Analysis on the Clinical Cohort

Quality control analyses discarded 12/116 bile acids and lysophospholipids displaying more than 10% missing values or 10% outliers (according to the Tukey fences method, with alpha = 4 × Inter-quantile range). Next, multiple imputation of missing values was performed using chained equations [30]. Multivariate analyses were performed using sparse Partial Least Square Discriminant Analyses (sPLS-DA) and aimed to construct models to predict the responder/non-responder status. This class of method is suitable for high-dimensional analyses [31] (i.e., when the number of predictions, lipids, is larger than the number of subjects) and also when predictors show high levels of correlation among each other. Other methods, such as Random Forest or Gradient Boosting, are suitable for such analyses, yet those methods require a large sample size for reliable training and relative to sPLS-DA, detailed model interpretation remains more challenging [32].

Data were split randomly into a training set (corresponding to 75% of the data) and a test set (corresponding to the remaining 25% of the data). This corresponds to 76 subjects for training (38 responders and 38 non-responders), and 24 subjects for testing (12 responders and 12 non-responders).

Three different models were constructed:

- A clinical model based solely on baseline clinical parameters, including total lipid levels (triglycerides, HDL, cholesterol, and LDL), the Body Mass Index (BMI), homeostasis model assessment of insulin resistance (HOMA-IR), gender, and age;
- A model consisting of only baseline bile acid and lysophoslipid levels;
- A model consisting of only the log2 fold-change that occurred during weight loss in bile acids and lysophoslipids.

Model training and evaluation were performed using the mixOmics R package [33]. The sPLS-DA models were constructed on the training set, using internal five-fold cross-validation. This led to the identification of the optimal parameters (i.e., optimal number of components and optimal number of variables per component) to build the model. Next, the final performance of the different trained models was assessed using the testing dataset (not used for model training).

An analysis of the change in lipid levels that occurred during the intervention was performed using a linear mixed effects model, with an interaction term between time and the subjects’ group (responders vs. non-responders). Additional covariates were included to adjust for age and gender (as fixed effects) and investigation sites and subject identifiers (as random effects). Post-hoc analyses were performed using the Tukey Honest Significant Difference approach. All analyses were performed using R statistical language (version 3.4.4).

## 3. Results and Discussion

### 3.1. Method Development and Validation

Good chromatographic separation of the different bile acid isomers is critical for their accurate quantification by mass spectrometry. For that reason, three different stationary phases (Waters HSS T3, BEH C8, and BEH Shield RP18) were evaluated during method development. A water-methanol system containing an acetic acid/ammonium acetate buffer was chosen as the eluent, as it is known to be well-suited to the LC-MS quantification of bile acids [16]. In order to be applicable to the analysis of large-scale clinical studies, the method duration was kept as short as possible. After optimization, a 13-min linear gradient from 50% to 95% organic (total run time of 19 min) was selected as the best compromise between analyte separation and method throughput. While all three stationary phases allowed for the baseline separation of most bile acid species, only the BEH Shield RP18 was able to separate the three muricholic acid isomers, as well as their taurine conjugated forms (Figure S1). This column was thus selected for further method development and validation.

Bile acids are traditionally extracted from plasma by either protein precipitation or solid phase extraction [34]. During this method development, we decided to focus on protein precipitation as this method appeared to be better suited for application to a large set of samples. Three different solvents were tested (acetonitrile, acetonitrile +30 mM HCl, and methanol) while maintaining a fixed 1/10 ratio between sample and precipitation agent volumes. The results for all analytes are summarized in Table S2. Recoveries were calculated after protein precipitation with an acetonitrile range from 13.3 to 102.1%. The addition of HCl to acetonitrile reduced the recovery of most analytes. In comparison, protein precipitation with methanol gave much better results. As all recoveries were within an acceptable range (84.4–99.2%), this procedure was selected for the next steps of method validation.

Once chromatographic separation and sample preparation procedure were optimized, the analytical performances of the method were tested. First, 8-point calibration curves for each bile acid were created and their linearity evaluated. In order to quantify circulating levels of bile acids, calibration curves were designed to cover the approximate concentrations of 0.003–5 µM. Two sets of calibration curves were prepared: one in HPLC solvent and the second in charcoal stripped plasma. In both cases, the linearity was good (R^2^ > 0.995) and slopes were comparable (Table S3). This indicates a limited matrix effect and opens up the possibility for quantifying plasma bile acids using calibration curves built in solvent. The only exceptions are the three TMCA isomers for which a clear matrix effect was observed. The three analytes were removed for the next steps of method validation.

The reproducibility, accuracy, precision, and dynamic range (linearity, limit of detection, and quantification) were evaluated for the 26 remaining bile acids. To assess dynamic range and lower limit of quantification (LLOQ) of the method, we performed 8-point calibrations for every bile acid using triplicates for each calibration level. The linearity of the obtained calibration curves was assessed in the range of 0.003−5.000 μM. A coefficient of determination (R^2^) > 0.995 was calculated for all bile acids. LOQs were in the range of 0.003–0.009 µM and LOD was within 0.0002–0.001 µM. Detailed results are shown in Table S4.

The precision and accuracy of the method were assessed by performing a triplicate analysis of spiked plasma samples at three different concentrations (0.02, 0.20, and 2.00 µM) on six different days. Precision, calculated as the relative standard deviation of calculated amounts, was generally within 10% for all analytes and levels. An exception was GLCA spiked at a low concentration (48.5%). The low precision measured for GLCA can be explained by the poor shape of the GLCA signal, which is due to a lack of mass accuracy. When higher amounts of analyte were spiked, the signals displayed a Gaussian shape and the precision was higher (101.1 and 94.4%), suggesting that a co-eluting isobaric impurity could be responsible for the lower mass accuracy. An examination of blank (non-spiked) plasma samples confirmed this hypothesis by revealing the presence of a co-eluting signal at *m*/*z* 432.3213, which is the M+1 signal of *m*/*z* 431.3181. For the same reason, the measured accuracy for GLCA spiked at a low concentration was poor (61.0%). All other levels and analytes gave much better results (86.8–112.1%). Detailed results are shown in Table S5.

In addition to the quantification of known bile acids, the method offers the possibility to screen for unknown bile acid species. Unknown species can be characterized based on their exact mass, but information collected in the all-ion fragmentation scan can also be helpful for compound identification. This is especially true for conjugated bile acids whose fragmentation patterns are characteristic [20]. Glycine conjugated bile acids show a C_3_H_4_NO_2_^−^ fragment at *m*/*z* 74.0248, whereas taurine conjugated bile acids produce multiple fragments at *m*/*z* 79.9574 (O_3_S^−^), 106.9808 (C_2_H_3_O_3_S^−^), and 124.0074 (C_2_H_6_NO_3_S^−^). The fragmentation of sulfated bile acids results in the loss of a neutral SO_3_ fragment (*m*/*z* 79.9597), but also in the production of an HO_4_S^−^ fragment at *m*/*z* 96.9601. Bile acids can also be conjugated to a glucuronide group [35]. For these species, the presence of a glucuronide fragment (C_6_H_9_O_7_^−^, *m*/*z* 193.0354) was expected.

**Figure 1:**
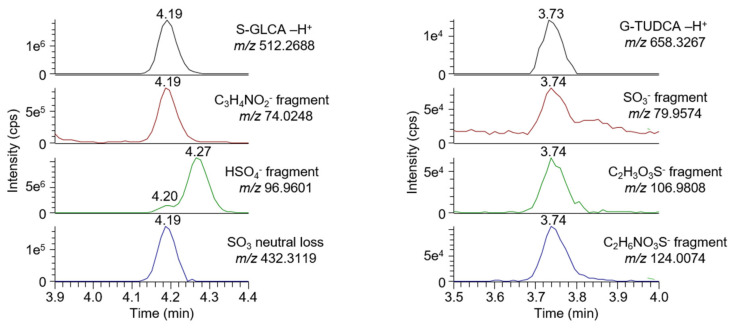
Chromatographic traces of S-GLCA (**left**) and G-TUDCA (**right**). The signal detected on the MS1-extracted chromatogram (**top**) co-eluted with characteristic fragments from the conjugated glycine and taurine, respectively.

To test the feasibility of the approach, we performed a screening experiment on a commercial sample of human plasma. The co-elution of characteristic fragments with the MS^1^ extracted chromatogram signal was used to help identify bile acids, as shown with the two examples of Figure 1. Because bile acids are often positional isomers, a complete and unambiguous signal assignment is only possible by the injection of a matching standard. In our preliminary screening experiment, twelve signals were detected (Table S6), which were screened in our clinical cohort.

**Figure 2:**
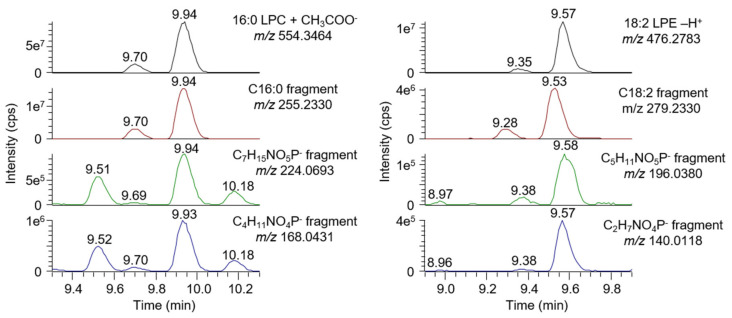
Extracted chromatograms of 16:0 lysophosphatidylcholines (LPC) (**top left**) and 18:2 lysophosphatidylethanol (LPE) (**top right**) in plasma. Two signals corresponding to 2-16:0 LPC (9.94 min) and 1-16:0 LPC (9.70 min) were detected. Fatty acid and head group fragments co-eluted with the molecular ions, thus confirming their identity. Both 18:2 LPE signals (2-18:2 LPE at 9.35 min and 2-18:2 LPE at 9.57 min) co-eluted with head group fragments. The main signals detected in the C18:2 fragment-extracted chromatogram (at 9.28 and 9.53 min) resulted from the 18:2 LPC.

The screening of plasma samples also revealed multiple well-defined signals between 9–12 min and *m*/*z* 500–600. An inspection of MS^2^ spectra revealed the presence of abundant signals corresponding to fatty acids such as palmitic, steric, oleic, and linoleic acid, as well as typical fragments of the phospholipid head group, particularly phosphocholine (as shown in the example of Figure 2) [36]. In an attempt to assign these signals, we injected mixtures of lysophospholipids (LPL), which were the most likely candidates to match the mass range of detected signals. We found multiple matching signals: Mostly lysophosphatidylcholine (LPC), but also lysophosphatidylethanol (LPE), lysophosphatidylinositiol (LPI), lysophosphatidylserine (LPS), and lysophosphatidylglycerol (LPG). The deprotonated forms of LPE, LPI, LPS, and LPG were detected, whereas the acetate adduct of LPC was detected. In each extracted chromatogram, a pair of signals separated by about 0.3 min and with an approximate 1:10 ratio was observed. Each signal likely corresponds to one of the two LPL isomers (1- vs. 2-LPL). The measured abundance ratio between signals also matched the standards’ specifications (predominantly the 1-LPL isomer, with up to 10% 2-LPL). To eliminate any ambiguity, we injected a 2-18:0 LPC standard, first individually and then together with 1-18:0 LPC. The results were as predicted, with the 2-isomer being separated from the 1-isomer and eluting first. All tested standards, as well as their detection parameters, are summarized in Table S7.

Recovery of extraction was tested for all lysophospholipid standards. The recovery was good for all of the LPL and within the accepted range (70–120%) (Table S7). The response linearity was tested in the range of reported LPL physiological concentrations [37,38]. A good linearity was obtained for all tested standards (R^2^ > 0.995) in the approximate range of 0.01–10 µM.

**Figure 3:**
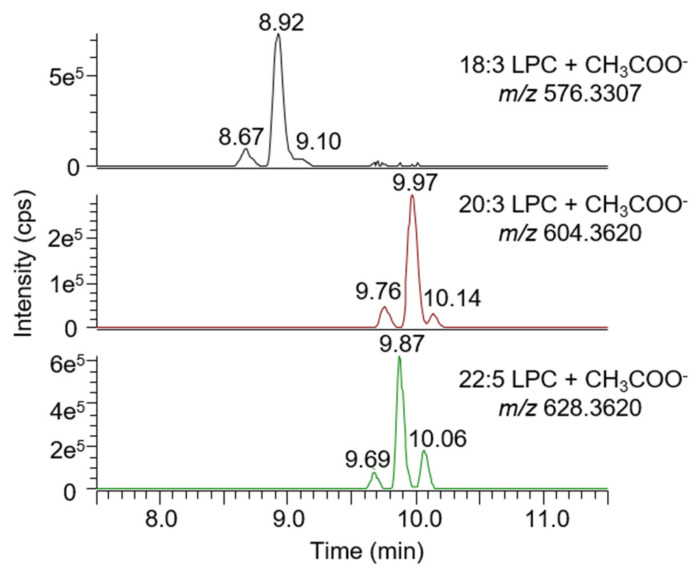
Extracted chromatograms of 18:3 LPC (**top**), 20:3 LPC (**middle**), and 22:5 LPC (**bottom**) in plasma. In all cases, a third signal could be observed in addition to the classical sn-1/sn-2 pair of signals. A similar pattern was observed for LPE and lysophosphatidylinositiol (LPI).

The reproducibility of the lysophospholipids detection was evaluated by analyzing six plasma samples from a healthy, fasted donor on six different days (n = 36). In these samples, 71 LPL could be consistently detected and tentatively identified based on their exact mass and retention time predictions. Among these LPL, 24 were LPC, 4 O-LPC, 17 LPE, 12 LPI, 9 LPG, and 5 LPS with a fatty acyl side chain ranging from C12:0 to C20:0. All of them were 1-LPC, although in most cases, a signal corresponding to 2-LPL could be detected. Fifteen of them (7 LPC, 5 LPE, and 3 LPI) were also included in the reproducibility assessment. For LPC, LPE, and LPI having a polyunsaturated fatty acyl side chain and thus several possible isomers, multiple signals could be detected on the extracted chromatograms (Figure S2). For C20:3 and C22:5, two baseline separated signals could be integrated, whereas for C18:3, signal shouldering could be observed (Figure 3).

**Figure 4:**
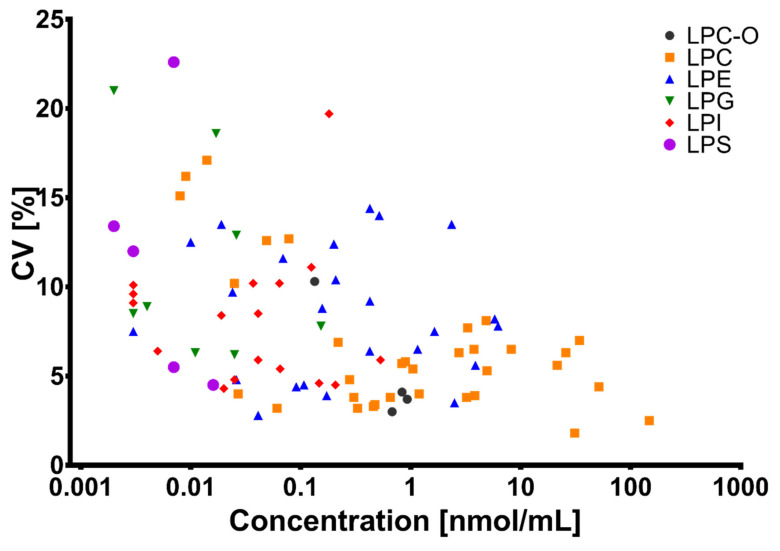
Correlation between the coefficient of variation and average lysophospholipid concentration in a set of 36 plasma samples analyzed on six different days.

The concentration of all 92 LPL species was estimated using internal standard amounts and the results were used for reproducibility calculations (Table S8). Estimated concentrations were in the range of 0.002–150 nmol/mL, with LPC, LPE, and O-LPC generally having higher concentrations than LPI, LPG, and LPS. The reproducibility of the measurements was good, with only two LPL having a coefficient of variation over 20% and five between 15 and 20%. Among these seven lipids, all but one had low concentrations (<0.020 nmol/mL), which is in line with the inverse correlation observed between the lipid concentration and coefficient of variation in this data set (Figure 4) and others [39,40]. The identity of lysophospholipids was confirmed with either commercially available standards or, in case none of them were available, fatty acid and/or head group fragments. The identity of 83 out of the 92 LPL detected in our plasma sample could be confirmed with at least one of these criteria. No characteristic fragments could be detected for the remaining six LPL, presumably due to their low concentration (<0.010 nmol/mL). Taking into consideration the two aspects discussed above, namely the reproducibility of the detection and identity confirmation with characteristic fragments, the limit of detection of our method can be estimated to be around 0.020 nmol/mL for lysophospholipids.

### 3.2. Application of the High Throughput Screening Method for Biomarker Discovery: Predicting the Response to a Controlled Weight Loss Intervention

We aimed to evaluate and validate the benefit of our validated method for biomarker discovery in obese non-diabetic patients. We thus quantified lysophospholipids and bile acids from plasma samples of 100 overweight/ obese subjects from the DiOGenes study [26] (results shown in Table 2). This intervention consisted of an 8-week low-caloric diet (LCD), followed by a 6-month weight maintenance phase. We previously classified subjects as being responders or non-responders at the end of the two phases [28]. Subjects from both groups had achieved >8% weight loss during LCD, yet only the responders (~50% of the population) achieved significant improvements in glycemic control, therefore reducing their risk of developing further metabolic complications, such as type 2 diabetes. The identification of obese subjects that do not improve their metabolic parameters despite strong weight loss remains a poorly understood clinical issue despite very recent improvements [41]. Here, we evaluated whether a signature could be identified from circulating bile acids and LPL and could provide a robust identification of non-responders.

**Figure 5:**
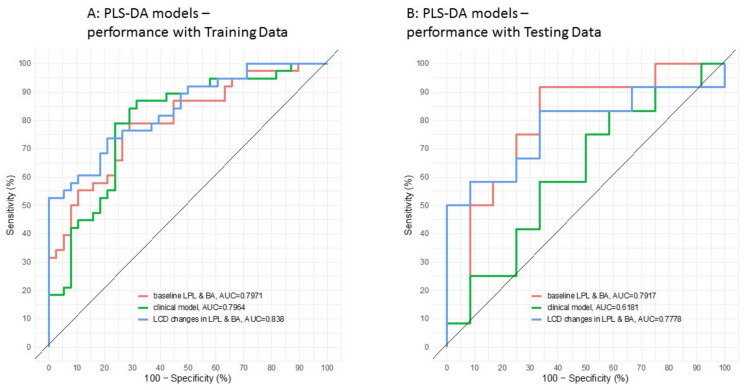
Receiving Operating Curves (ROC) used to predict weight loss responders and non-responders. (**A**) Performance for the training set, and (**B**) performance for the testing set.

We first constructed a clinical model based on parameters that are easily available in a clinical routine, namely the body mass index (BMI), age, gender, HOMA-IR (a measure of insulin resistance), and total lipids (cholesterol, HDL, LDL, and triglycerides). While the trained clinical model achieved a good performance (AUC = 79.64%, Figure 5A), it only performed modestly for the independent testing set (with AUC = 61.81%, Figure 5B). Then, we constructed two models, solely based on lysophospholipids and bile acids; the first model consisted of baseline levels, while the second model used the fold-change in lipid levels during the weight loss phase. Both trained models reached a good performance (AUCs close to 80%, Figure 5A), and exhibited a similar performance with the testing dataset (Figure 5B). The model based on baseline lipid profiles had a slightly higher performance than the model using lipid fold-change during the intervention (ROC AUCs 79.17% vs. 77.78%), and these two models clearly outperformed the clinical model, which only reached AUC 61.81%. In a clinical setting, the model with baseline lipid profiles would be preferred owing to its simplicity (compared to the model based on two timepoints: Before and after weight loss).

**Figure 6:**
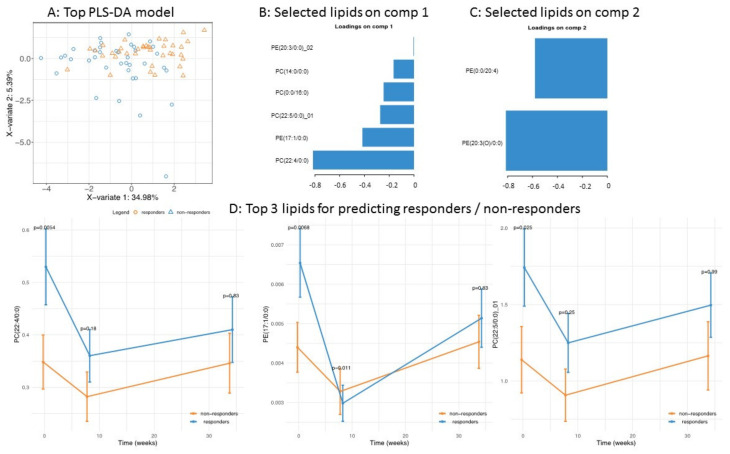
Top model was employed to predict weight loss responders and non-responders: (**A**) Score plot separating responders and non-responders; (**B**,**C**) lipids contributing to the prediction of PC1 and PC2; (**D**) time-series profile (during weight loss intervention) of the top three lipids in the PLS-DA model.

Figure 6A displays the separation between responders and non-responders based on their baseline lipid profiles and indicates that the first component of the statistical model (sPLS-DA), which already captures most of the data variance (35%); the second component only represents a modest improvement (representing about 5% of the variance). Figure 6B,C displays the lipids that are driving these two components, and Figure 6D shows the evolution of the top three lipids for all timepoints in the clinical study. Interestingly, for all three lipids (PC(22:4/0:0), PE(17:1/0:0), and PC(22:5/0:0)), the responder group started with significantly higher baseline levels relative to the non-responder group. Additional analyses comparing changes in lipid levels during the intervention (see Methods) showed that, in the responders, all three lipids had significantly lower levels following weight loss (Table S9) and that for two of them (PC(22:4/0:0) and PE(17:1/0:0)), their levels at study termination (after the 6-month weight maintenance) remained significantly lower than the baseline levels (Table S9). Conversely, non-responders only showed a reduction in PC(22:5/0:0)_01 during the weight loss phase, but this change did not persist during the weight maintenance phase.

While the role of lysophosphatidylcholines (LPC) has been studied in the context of obesity and weight loss [1,42,43], little is known about the identified species, due to the lack of a quantitative method that targets the wide range of these molecular species. In mice, levels of PC(22:4/0:0) were increased upon the introduction of a high-fat diet and correlated with total cholesterol [43]. In humans [42], LPC 22:4 displayed a marginal association (*p* = 0.065) with the change in BMI during a low-caloric intervention in 57 obese subjects. Similarly, PC(17:1/0:0) is not frequently quantified in LPC studies, yet a marginal association (*p* = 0.095) could be found following weight loss in 33 subjects suffering from non-alcoholic fatty liver disease [44]. To the best of our knowledge, PC(22:5/0:0) has not previously been reported to be associated with weight loss.

### 3.3. Application of the High Throughput Screening Method for Biomarker Discovery: Predicting Insulin Resistance

**Figure 7:**
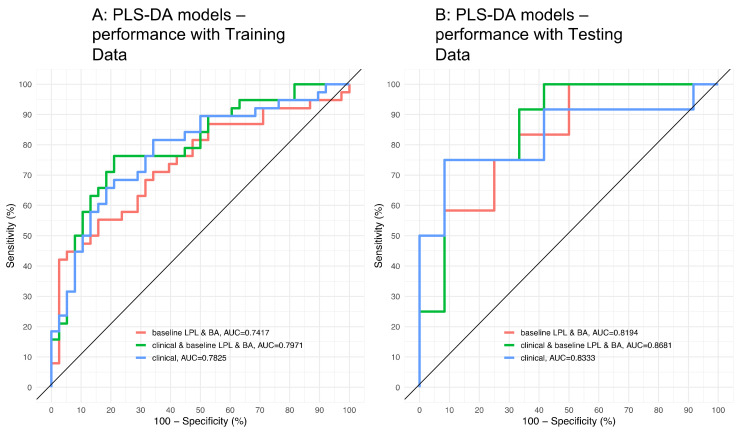
Receiving Operating Curves (ROC) used to predict subjects with high/low homeostasis model assessment of insulin resistance (HOMA-IR): (**A**) Performance for the training set, and (**B**) performance for the testing set.

Here, we aimed to predict a high degree of insulin resistance (HOMA-IR > 3) in the same population of overweight/obese subjects, prior to any intervention. A first model (“clinical model”) based solely on BMI, age, gender, and total lipid levels achieved a relatively good performance (for the testing set, ROC AUC = 83.33%). A lipidomics-based model achieved a slightly lower, though comparable performance (AUC = 81.94%). Interestingly, a more complex model blending clinical parameters and lipidomics improved the performance (AUC = 86.81%, see Figure 7).

**Figure 8:**
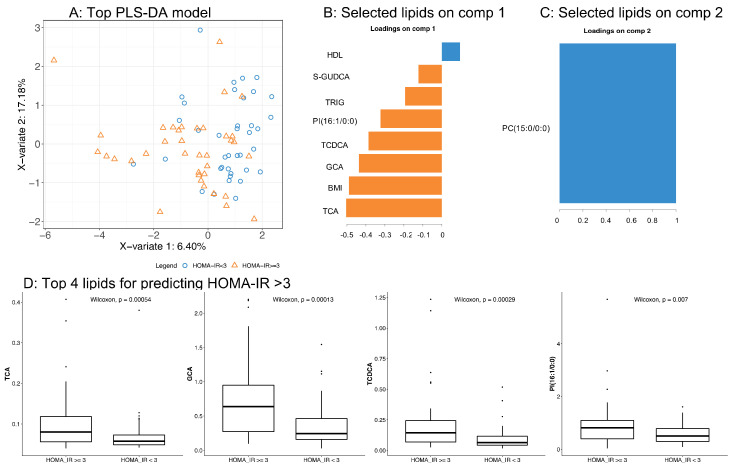
Top model was employed to predict subjects with high/low HOMA-IR: (**A**) Score plot separating responders and non-responders; (**B**,**C**) key parameters contributing to the prediction of PC1 and PC2; and (**D**) baseline levels of the top four lipids in the PLS-DA model.

In this model, the top five predictors of insulin resistance were TCA, BMI, GCA, TCDCA, and PI(16:1/0:0) (see Figure 8); for all of them, increased levels were associated with an increased likelihood of having a high insulin resistance (see Figure 8D). Our findings are in agreement with previous reports on the positive association between total bile acids, including TCA and GCA, and HOMA-IR in obese subjects [45]. Kalhan et al. reported a significant increase in the fasting plasma concentration of bile acids, including glycocholate, taurocholate, and taurochenodeoxycholate, in patients with nonalcoholic Steatohepatitis (NASH) compared to controls [46], while Ferslew et al. observed an increase in the levels of both primary and secondary bile acids in patients with NASH [47]. However, the association with insulin resistance was not investigated in these studies.

Although there are several reports on BA and LPL as potential biomarkers, very few have been validated and used in clinical practice. While, in general, consistent results have been obtained across many studies in association with metabolic disorders, the candidate biomarker signatures reported are different [48]. This is, in part, due to the application of different methods that, in some cases, especially an untargeted metabolomics approach, have not been fully validated for these molecular species. In other cases, these different biomarkers may be explained by a non-comprehensive analysis of bile acids [49] or a lack of chromatographic separation between BA isomers [50].

## 4. Conclusions

In conclusion, we propose a screening method that enables the profiling of both bile acids and LPL in plasma, with an accurate precision and a throughput that makes it very attractive for applications in large clinical cohorts and eventually routine patient monitoring at the hospital. We demonstrated the application of our method for biomarker discovery with the analysis of obese subjects from the DiOGenes study. We identified relevant markers predicting a successful response to a low-caloric diet intervention in terms of both weight loss and glycemic control improvements. To the best of our knowledge, these markers have not been previously identified, partly due to a lack of comprehensive measurements of these lipid species in clinical cohorts. Within the few studies that quantified these molecular species, only marginal associations with clinical outcome were found. This might be due to a slightly smaller sample size (n ranging from 33 to 37 subjects, while our discovery approach used 75 subjects), and the use of more powerful statistical methods (multivariate vs, classical *t*-tests), together with better control of the technical variability obtained from our method.

## Figures and Tables

**Table 1:** Baseline characteristics of selected patients. Numbers represent the mean and standard deviation. For gender, the *p*-value was obtained from a two-sided Fisher’s exact test; for all other parameters, the *p*-value was obtained from a two-sided *t*-test. All parameters are representative of the whole DiOGenes cohort, including recent observations made from deep phenotyping analyses of 174 responders and 201 non-responders [29].

Parameter	All Subjects (N = 100)	Non Responders (N = 50)	Responders (N = 50)	*p*-Value
age, y	42+/−6	41+/−6	43+/−6	0.0423
BMI, kg/2	35.2703+/−5.1284	36.0502+/−5.3902	34.4904+/−4.7794	0.129
fasting cholesterol levels, mmol/L	4.6770+/−0.9588	4.5218+/−0.8869	4.8323+/−1.0107	0.106
HOMA-IR	3.4239+/−1.8902	3.2890+/−2.1510	3.5588+/−1.5983	0.478
fasting LDL, mmol/L	2.8845+/−0.8245	2.7662+/−0.7372	3.0053+/−0.8964	0.151
gender	M = 44, F = 56	M = 14, F = 36	M = 30, F = 20	0.002333
fasting HDL, mmol/L	1.1104+/−0.3039	1.2044+/−0.3291	1.0164+/−0.2453	0.00168
fasting triglycerides, mmol/L	1.4660+/−0.6817	1.2244+/−0.5851	1.7124+/−0.6904	0.000265

**Table 2:** Levels of circulating bile acids measured in the DiOGenes cohort. Results are expressed as the median (10th–90th percentiles).

Symbol	Bile Acid	Concentration (µM)
CA	Cholic acid	0.132 (0.069–0.976)
CDCA	Chenodeoxycholic acid	0.265 (0.058–2.027)
DCA	Deoxycholic acid	1.221 (0.208–3.697)
LCA	Lithocholic acid	0.125 (0.038–0.318)
UDCA	Ursodeoxycholic acid	0.728 (0.210–3.343)
HCA	Hyocholic acid	0.016 (0.008–0.042)
HDCA	Hyodeoxycholic acid	0.075 (0.025–0.185)
GCA	Glycocholic acid	0.282 (0.095–0.955)
GCDCA	Glycochenodeoxycholic acid	1.445 (0.406–4.155)
GDCA	Glycodeoxycholic acid	0.252 (0.060–1.017)
GLCA	Glycolithocholic acid	ND
GUDCA	Glycoursodeoxycholic acid	0.113 (0.060–0.387)
GHDCA	Glycohyodeoxycholic acid	ND
TCA	Taurocholic acid	0.058 (0.044–0.129)
TCDCA	Taurochenodeoxycholic acid	0.081 (0.026–0.270)
TDCA	Taurodeoxycholic acid	0.033 (0.014–0.109)
TLCA	Taurolithocholic acid	0.022 (0.009–0.042)
TUDCA	Tauroursodeoxycholic acid	0.025 (0.016–0.246)
THDCA	Taurohyodeoxycholic acid	ND
α-MCA	α-muricholic acid	0.015 (0.008–0.031)
β-MCA	β-muricholic acid	ND
ω-MCA	ω-muricholic acid	0.027 (0.012–0.074)
7S-CA	Cholic acid 7-sulfate	ND
3S-TLCA	Taurolitocholic acid 3-sulfate	0.055 (0.016–0.174)
3S-TCA	Taurocholic acid 3-sulfate	ND
MDCA	Murideoxycholic acid	ND

## Supplementary Materials

The following are available online at https://www.mdpi.com/2218-273X/10/7/1049/s1: Table S1: List of bile acid standards used for method development and validation; Table S2: Recoveries ± SD calculated for the three tested protein precipitation techniques; Table S3: Plasma and solvent calibration curve parameters; Table S4: Method validation parameters: Limit of detection, dynamic range, and linearity; Table S5: Method validation parameters for bile acids; Table S6: List of bile acids detected during untargeted screening of a plasma sample; Table S7: List of lysophospholipid standards with their detection parameters; Table S8: List of lysophospholipids detected in plasma during the validation experiment; Table S9: Linear mixed effects model, post-hoc analyses (Tukey’s HSD); Figure S1: Extracted chromatograms of bile acids spiked in a plasma sample; Figure S2: Extracted chromatograms of lysophospholipids in a plasma sample.
