# Supplementary material for: Untargeted Profiling of Bile Acids and Lysophospholipids Identifies the Lipid Signature Associated with Glycemic Outcome in an Obese Non-Diabetic Clinical Cohort
Source: Biomolecules. 2020 Jul 15;10(7):1049. doi: 10.3390/biom10071049 (PMC7407211; doi:10.3390/biom10071049)
Supplement: Supplementary file 1 [file biomolecules-10-01049-s001.pdf]

Supporting Information

Untargeted Profiling of Bile Acids and Lysophospholipids Identifies Lipid Signature Associated to Glycemic Outcome in Obese Non-Diabetic Clinical Cohort

Nicolas Christinat[1], Armand Valsesia [1], and Mojgan Masoodi[1,2*]

[1] Nestlé Research, Nestlé Institute of Health Sciences, Lausanne, Switzerland

[2] Institute of Clinical Chemistry, Inselspital, Bern University Hospital, Bern, Switzerland

Table of Content

Table S1: List of bile acids standards used for method development and validation.

Table S2: Recoveries ± SD calculated for the three tested protein precipitation techniques.

Table S3: Plasma and solvent calibration curves parameters.

Table S4: Method validation parameters: limit of detection, dynamic range and linearity.

Table S5: Method validation parameters for bile acids.

Table S6: List of bile acids detected during untargeted screening of a plasma sample.

Table S7: List of lysophospholipid standards with their detection parameters.

Table S8: List of lysophospholipid detected in plasma during validation experiment.

Table S9: Linear mixed effect model, post-hoc analyses (Tukey's HSD).

Figure S1: Extracted chromatograms of bile acids spiked in a plasma sample.

Figure S2: Extracted chromatograms of lysophospholipids in a plasma sample.

Symbol	Bile acid	Chemical formula	CAS number	Exact mass [-H$^+$]	RT [min]
CA	Cholic acid	$C_{24}H_{40}O_5$	81-25-4	407.2803	5.26
CDCA	Chenodeoxycholic acid	$C_{24}H_{40}O_4$	474-25-9	391.2854	7.03
DCA	Deoxycholic acid	$C_{24}H_{40}O_4$	83-44-3	391.2854	7.14
LCA	Lithocholic acid	$C_{24}H_{40}O_3$	434-13-9	375.2905	8.87
UDCA	Ursodeoxycholic acid	$C_{24}H_{40}O_4$	128-13-2	391.2854	5.17
HCA	Hyocholic acid	$C_{24}H_{40}O_5$	547-75-1	407.2803	4.91
HDCA	Hyodeoxycholic acid	$C_{24}H_{40}O_4$	83-49-8	391.2854	5.64
GCA	Glycocholic acid	$C_{26}H_{43}NO_6$	475-31-0	464.3018	3.97
GCDCA	Glycochenodeoxycholic acid	$C_{26}H_{43}NO_5$	640-79-9	448.3069	5.61
GDCA	Glycodeoxycholic acid	$C_{26}H_{43}NO_5$	360-65-6	448.3069	5.92
GLCA	Glycolithocholic acid	$C_{26}H_{43}NO_4$	474-74-8	432.3119	7.43
GUDCA	Glycoursodeoxycholic acid	$C_{26}H_{43}NO_5$	64480-66-6	448.3069	3.43
GHDCA	Glycohyodeoxycholic acid	$C_{26}H_{43}NO_5$	13042-33-6	448.3069	3.77
TCA	Taurocholic acid	$C_{26}H_{45}NO_7S$	81-24-3	514.2844	3.90
TCDCA	Taurochenodeoxycholic acid	$C_{26}H_{45}NO_6S$	516-35-8	498.2895	5.56
TDCA	Taurodeoxycholic acid	$C_{26}H_{45}NO_6S$	516-50-7	498.2895	5.83
TLCA	Taurolithocholic acid	$C_{26}H_{45}NO_5S$	516-90-5	482.2946	7.33
TUDCA	Tauroursodeoxycholic acid	$C_{26}H_{45}NO_6S$	14605-22-2	498.2895	3.39
THDCA	Taurohyodeoxycholic acid	$C_{26}H_{45}NO_6S$	38411-85-7	498.2895	3.73
α-MCA	α-muricholic acid	$C_{24}H_{40}O_5$	2393-58-0	407.2803	3.92
β-MCA	β-muricholic acid	$C_{24}H_{40}O_5$	2393-59-1	407.2803	4.22
ω-MCA	ω-muricholic acid	$C_{24}H_{40}O_5$	6830-03-1	407.2803	3.75
α-TMCA	Tauro-α-muricholic acid	$C_{26}H_{45}NO_7S$	25613-05-2	514.2844	1.97
β-TMCA	Tauro-β-muricholic acid	$C_{26}H_{45}NO_7S$	25696-60-0	514.2844	2.11
ω-TMCA	Tauro-ω-muricholic acid	$C_{26}H_{45}NO_7S$	130325-58-5	514.2844	1.87
7S-CA	Cholic acid 7-sulfate	$C_{24}H_{40}O_8S$	60320-05-0	487.2371	3.16
3S-TLCA	Taurolitocholic acid 3-sulfate	$C_{26}H_{45}NO_8S_2$	64939-83-0	562.2514	4.26
3S-TCA	Taurocholic acid 3-sulfate	$C_{26}H_{45}NO_{10}S_2$	67030-62-0	594.2412	1.39
MDCA	Murideoxycholic acid	$C_{24}H_{40}O_4$	668-49-5	391.2854	4.96

Table S1: List of bile acids standards used for method development and validation.

Bile acid*	Acetonitrile [%]	Acetonitrile + 30 mM Hydrochloric acid [%]	Methanol [%]
3S-TCA	45.7 ± 2.3	7.6 ± 0.3	91.9 ± 2.2
α-TMCA	87.2 ± 2.8	51.5 ± 2.1	97.0 ± 2.0
ß-TMCA	93.9 ± 3.0	5.1 ± 0.5	99.2 ± 2.1
ω-TMCA	57.4 ± 3.1	37.2 ± 3.4	87.7 ± 0.6
7S-CA	13.3 ± 1.0	55.1 ± 2.1	90.9 ± 2.3
TUDCA	97.4 ± 4.0	75.0 ± 0.9	93.5 ± 2.0
GUDCA	83.0 ± 5.3	94.8 ± 2.0	94.7 ± 1.1
THDCA	90.5 ± 4.8	65.0 ± 0.8	91.8 ± 0.3
ω-MCA	38.7 ± 2.5	69.0 ± 0.7	95.2 ± 0.6
GHDCA	64.2 ± 3.1	93.3 ± 0.7	93.6 ± 0.9
TCA	69.2 ± 4.7	32.1 ± 1.9	88.8 ± 6.4
α-MCA	47.2 ± 3.7	87.2 ± 2.8	95.2 ± 0.6
GCA	43.9 ± 3.0	65.4 ± 1.3	88.3 ± 1.7
ß-MCA	54.7 ± 4.2	21.7 ± 0.5	95.4 ± 2.0
3S-TLCA	79.0 ± 4.1	24.9 ± 0.3	89.5 ± 3.6
HCA	22.3 ± 1.3	82.2 ± 4.3	95.0 ± 0.2
MDCA	77.4 ± 4.4	96.6 ± 2.1	94.8 ± 0.4
UDCA	75.3 ± 2.5	96.3 ± 2.2	93.1 ± 0.9
CA	49.6 ± 2.4	82.9 ± 2.0	91.0 ± 0.5
TCDCA	90.2 ± 4.3	57.5 ± 0.4	84.7 ± 3.9
GCDCA	72.8 ± 3.8	85.6 ± 1.5	86.5 ± 3.6
HDCA	51.9 ± 2.4	92.8 ± 1.0	92.1 ± 0.8
TDCA	97.3 ± 4.1	64.2 ± 3.6	84.6 ± 3.4
GDCA	69.9 ± 3.8	90.0 ± 2.4	86.1 ± 4.6
CDCA	66.8 ± 4.5	84.2 ± 2.9	87.1 ± 1.3
DCA	75.1 ± 3.5	85.1 ± 2.4	86.6 ± 1.2
TLCA	102.1 ± 6.1	80.9 ± 0.6	86.4 ± 1.6
GLCA	91.8 ± 5.4	81.1 ± 3.7	84.4 ± 2.4
LCA	90.3 ± 5.1	64.8 ± 5.7	85.9 ± 5.6

*Bile acids abbreviations are listed in table S1.

Table S2: Recoveries ± SD calculated for the three tested protein precipitation techniques.

Bile acid*	Solvent		Human plasma		Slope ratio
	Equation	R^2	Equation	R^2	Plasma/solvent
3S-TCA	y = 0.2507x - 0.0002	0.9996	y = 0.2648x + 0.0001	0.9993	1.056
ω-TMCA	y = 0.4691x + 0.0003	0.9994	y = 0.3988x - 0.0020	0.9970	0.850
α-TMCA	y = 4.8171x + 0.0110	0.9981	y = 3.5667x + 0.0022	0.9971	0.740
ß-TMCA	y = 2.3797x - 0.0008	0.9999	y = 2.1804x - 0.0048	0.9967	0.916
7S-CA	y = 0.8306x - 0.0002	0.9992	y = 0.8768x + 0.0001	0.9997	1.056
TUDCA	y = 6.2618x - 0.0089	0.9984	y = 6.5000x - 0.0099	0.9979	1.038
GUDCA	y = 4.4270x + 0.0270	0.9980	y = 4.3336x + 0.0264	0.9993	0.979
THDCA	y = 5.7928x - 0.0097	0.9991	y = 6.0413x - 0.0101	0.9975	1.043
ω-MCA	y = 3.0443x - 0.0065	0.9995	y = 3.3281x + 0.0008	0.9991	1.093
GHDCA	y = 4.6495x -0.0024	0.9995	y = 4.6477x - 0.0021	0.9986	1.000
TCA	y = 3.4102x - 0.0012	0.9993	y = 3.3708x - 0.0017	0.9985	0.988
α-MCA	y = 2.2504x - 0.0027	0.9997	y = 2.1430x - 0.0021	0.9992	0.952
GCA	y = 5.4964x - 0.0006	0.9976	y = 5.3388x + 0.0012	0.9992	0.971
ß-MCA	y = 4.0368x - 0.0064	0.9991	y = 3.8448x - 0.0052	0.9981	0.952
3S-TLCA	y = 1.5561x - 0.0002	0.9987	y = 1.5368x + 0.0033	0.9997	0.988
HCA	y =2.4988x - 0.0005	0.9996	y = 2.5455x - 0.0015	0.9997	1.019
MDCA	y = 10.3675x + 0.0001	0.9978	y = 10.3777x - 0.0018	0.9999	1.001
UDCA	y = 8.7216x - 0.0057	0.9988	y = 8.7018x - 0.0042	0.9991	0.998
CA	y = 3.9539x + 0.0001	0.9990	y = 3.9443x - 0.0015	0.9993	0.998
TCDCA	y = 3.5670x - 0.0020	0.9980	y = 3.5798x - 0.0018	0.9983	1.004
GCDCA	y = 7.5823x + 0.0012	0.9988	y = 7.5597x + 0.0039	0.9992	0.997
HDCA	y = 3.8103x - 0.0021	0.9990	y = 3.7332x - 0.0011	0.9993	0.980
TDCA	y = 2.8849x - 0.0007	0.9988	y = 2.8995x - 0.0012	0.9984	1.005
GDCA	y = 3.1094x - 0.0007	0.9987	y = 3.0786x + 0.0032	0.9993	0.990
CDCA	y = 5.0424x + 0.0056	0.9993	y = 5.1585x + 0.0073	0.9994	1.023
DCA	y = 9.2886x - 0.0061	0.9984	y = 9.2635x -0.0049	0.9992	0.997
TLCA	y = 3.9372x + 0.0050	0.9990	y = 3.9468x + 0.0052	0.9987	1.002
GLCA	y = 5.4456x + 0.0009	0.9971	y = 5.7372x -0.0077	0.9991	1.054
LCA	y = 2.7444x - 0.0032	0.9987	y = 2.6974x -0.0005	0.9994	0.983

*Bile acids abbreviations are listed in table S1. R^2: coefficient of determination

Table S3: Plasma and solvent calibration curves parameters.

Bile acid*	LOD [µM]	Range [µM]	Slope	Intercept	coefficient of determination (R^2)
3S-TCA	0.001	0.003-0.750	0.320 ± 0.011	-0.0003 ± 0.0001	0.9994 ± 0.0004
ω-TMCA	0.003	0.009-2.500	0.654 ± 0.065	-0.0005 ± 0.0007	0.9974 ± 0.0013
α-TMCA	0.003	0.009-2.500	5.777 ± 0.492	0.0095 ± 0.0063	0.9960 ± 0.0034
ß-TMCA	0.0005	0.003-0.750	3.288 ± 0.426	-0.0033 ± 0.0032	0.9986 ± 0.0017
7S-CA	0.001	0.003-0.750	1.062 ± 0.053	-0.0004 ± 0.0004	0.9994 ± 0.0004
TUDCA	0.003	0.009-5.000	6.762 ± 0.226	-0.0129 ± 0.0038	0.9986 ± 0.0009
GUDCA	0.0002	0.003-5.000	5.105 ± 0.090	0.0260 ± 0.0033	0.9994 ± 0.0003
THDCA	0.003	0.009-5.000	7.396 ± 0.454	-0.0349 ± 0.0103	0.9982 ± 0.0009
ω-MCA	0.003	0.009-2.500	3.862 ± 0.201	-0.0072 ± 0.0040	0.9991 ± 0.0008
GHDCA	0.001	0.003-2.500	5.438 ± 0.141	-0.0049 ± 0.0029	0.9994 ± 0.0004
TCA	0.003	0.009-5.000	4.782 ± 0.148	-0.0143 ± 0.0080	0.9975 ± 0.0014
α-MCA	0.003	0.009-2.500	2.776 ± 0.097	-0.0044 ± 0.0033	0.9992 ± 0.0009
GCA	0.0005	0.003-5.000	7.493 ± 0.173	-0.0030 ± 0.0031	0.9994 ± 0.0003
ß-MCA	0.003	0.009-2.500	4.597 ± 0.237	-0.0029 ± 0.0014	0.9963 ± 0.0040
3S-TLCA	0.001	0.003-2.500	1.614 ± 0.201	0.0009 ± 0.0016	0.9956 ± 0.0018
HCA	0.003	0.009-2.500	3.131 ± 0.302	-0.0031 ± 0.0028	0.9995 ± 0.0004
MDCA	0.0003	0.003-2.500	13.828 ± 0.480	0.0001 ± 0.0029	0.9988 ± 0.0005
UDCA	0.0003	0.003-5.000	10.698 ± 0.159	-0.0079 ± 0.0038	0.9993 ± 0.0004
CA	0.003	0.003-5.000	5.530 ± 0.097	-0.0057 ± 0.0061	0.9994 ± 0.0003
TCDCA	0.001	0.003-5.000	4.626 ± 0.126	-0.0015 ± 0.0025	0.9990 ± 0.0005
GCDCA	0.0006	0.003-5.000	9.382 ± 0.542	0.0034 ± 0.0045	0.9993 ± 0.0003
HDCA	0.0003	0.003-5.000	4.276 ± 0.062	-0.0034 ± 0.0011	0.9994 ± 0.0002
TDCA	0.001	0.003-5.000	3.317 ± 0.075	-0.0033 ± 0.0038	0.9989 ± 0.0006
GDCA	0.001	0.003-5.000	4.764 ± 0.059	-0.0006 ± 0.0021	0.9996 ± 0.0003
CDCA	0.0003	0.003-5.000	6.878 ± 0.279	0.0012 ± 0.0019	0.9995 ± 0.0001
DCA	0.0003	0.003-5.000	11.079 ± 0.156	-0.0093 ± 0.0033	0.9992 ± 0.0003
TLCA	0.001	0.003-5.000	6.546 ± 0.218	0.0019 ± 0.0045	0.9992 ± 0.0004
GLCA	0.0003	0.003-2.500	7.896 ± 0.417	0.0050 ± 0.0034	0.9962 ± 0.0019
LCA	0.0003	0.003-5.000	8.015 ± 0.142	-0.0060 ± 0.0025	0.9992 ± 0.0002

*Bile acids abbreviations are listed in table S1. LOD: Limit of detection.

Table S4: Method validation parameters: limit of detection, dynamic range and linearity.

Bile acid*	Recovery [%]	Matrix Effect [%]	Nominal concentration [µM]	Measured concentration [µM]	Accuracy [%]	Precision [%]
3S-TCA	91.9 ± 2.2	104.7 ± 6.4	0.019	0.021 ± 0.001	110.3	2.5
			0.203	0.223 ± 0.007	109.7	3.0
			2.034	Out of linearity range		
7S-CA	90.9 ± 2.3	109.0 ± 6.9	0.019	0.020 ± 0.001	104.2	6.8
			0.200	0.214 ± 0.009	107.1	4.3
			1.997	Out of linearity range		
TUDCA	93.5 ± 2.0	103.1 ± 4.4	0.019	0.017 ± 0.001	92.8	3.8
			0.200	0.176 ± 0.005	88.1	2.6
			2.002	1.999 ± 0.059	99.9	2.9
GUDCA	94.7 ± 1.1	98.0 ± 3.4	0.019	0.018 ± 0.001	97.5	4.0
			0.200	0.188 ± 0.009	93.9	4.7
			2.000	1.940 ± 0.086	97.0	4.4
THDCA	91.8 ± 0.3	107.0 ± 7.1	0.019	0.020 ± 0.001	105.7	3.9
			0.200	0.180 ± 0.007	89.9	3.6
			2.000	2.241 ± 0.058	112.1	2.6
ω-MCA	95.2 ± 0.6	109.3 ± 5.0	0.019	0.017 ± 0.001	92.2	4.3
			0.200	0.174 ± 0.004	86.8	2.2
			2.002	1.893 ± 0.060	94.6	3.2
GHDCA	93.6 ± 0.9	100.4 ± 5.3	0.019	0.017 ± 0.001	93.1	4.8
			0.200	0.193 ± 0.009	96.3	4.9
			2.000	2.094 ± 0.069	104.7	3.3
TCA	88.8 ± 6.4	98.5 ± 5.7	0.019	0.021 ± 0.001	111.1	2.9
			0.200	0.179 ± 0.009	89.3	5.0
			2.000	2.071 ± 0.092	103.6	4.4
α-MCA	95.2 ± 0.6	98.6 ± 6.2	0.019	0.018 ± 0.001	97.5	5.7
			0.200	0.180 ± 0.010	90.1	5.4
			2.002	1.860 ± 0.068	92.9	3.7
GCA	88.3 ± 1.7	99.2 ± 5.6	0.019	0.018 ± 0.002	97.7	9.1
			0.200	0.185 ± 0.008	92.6	4.4
			2.000	1.826 ± 0.039	91.3	2.2
ß-MCA	95.4 ± 2.0	99.9 ± 8.0	0.019	0.017 ± 0.001	91.0	4.8
			0.200	0.183 ± 0.011	91.6	6.0
			2.000	1.855 ± 0.116	92.8	6.2
3S-TLCA	89.5 ± 3.6	99.7 ± 2.9	0.019	0.017 ± 0.003	90.3	14.8
			0.202	0.222 ± 0.011	110.3	4.7
			2.017	1.965 ± 0.101	97.4	5.2
HCA	95.0 ± 0.2	100.1 ± 8.3	0.019	0.017 ± 0.001	93.2	4.4
			0.200	0.186 ± 0.007	93.1	4.0
			1.998	1.938 ± 0.042	97.0	2.2
MDCA	94.8 ± 0.4	99.7 ± 5.6	0.019	0.017 ± 0.001	91.2	6.0
			0.200	0.195 ± 0.007	97.3	3.6
			2.002	1.929 ± 0.064	96.4	3.3

UDCA	93.1 ± 0.9	101.0 ± 5.5	0.019	0.018 ± 0.001	93.4	3.2
			0.200	0.183 ± 0.004	91.3	2.3
			2.002	1.934 ± 0.053	96.6	2.7
CA	91.0 ± 0.5	97.5 ± 5.8	0.019	0.018 ± 0.001	96.8	4.7
			0.199	0.186 ± 0.006	93.5	3.1
			1.993	1.952 ± 0.068	97.9	3.5
TCDCA	84.7 ± 3.9	100.6 ± 3.5	0.019	0.017 ± 0.001	93.2	4.4
			0.200	0.177 ± 0.004	88.6	2.0
			2.001	1.958 ± 0.046	97.8	2.4
GCDCA	86.5 ± 3.6	101.8 ± 6.1	0.019	0.020 ± 0.001	105.7	6.0
			0.200	0.191 ± 0.010	95.3	5.4
			2.002	1.980 ± 0.059	98.9	3.0
HDCA	92.1 ± 0.8	99.7 ± 4.6	0.019	0.017 ± 0.001	92.1	4.3
			0.200	0.182 ± 0.005	91.1	2.9
			2.001	1.913 ± 0.060	95.6	3.1
TDCA	84.6 ± 3.4	99.4 ± 4.1	0.019	0.018 ± 0.001	98.4	5.2
			0.200	0.181 ± 0.009	90.3	5.0
			2.001	1.992 ± 0.066	99.5	3.3
GDCA	86.1 ± 4.6	102.2 ± 7.7	0.019	0.020 ± 0.001	106.3	5.6
			0.200	0.188 ± 0.013	93.8	7.1
			2.001	1.901 ± 0.101	95.0	5.3
CDCA	87.1 ± 1.3	103.9 ± 5.6	0.019	0.018 ± 0.001	94.6	6.4
			0.200	0.186 ± 0.008	92.8	4.4
			2.002	1.956 ± 0.066	97.7	3.4
DCA	86.6 ± 1.2	100.6 ± 5.3	0.019	0.018 ± 0.001	96.4	5.3
			0.201	0.181 ± 0.011	90.5	5.8
			2.006	1.916 ± 0.055	95.5	2.9
TLCA	86.4 ± 1.6	100.3 ± 4.4	0.019	0.018 ± 0.001	95.4	5.6
			0.200	0.184 ± 0.004	91.9	2.3
			2.002	1.994 ± 0.063	99.6	3.2
GLCA	84.4 ± 2.4	101.1 ± 7.0	0.019	0.011 ± 0.006	61.0	48.5
			0.200	0.202 ± 0.013	101.1	6.3
			2.001	1.888 ± 0.171	94.4	9.1
LCA	85.9 ± 5.6	99.9 ± 6.5	0.019	0.019 ± 0.001	99.9	5.0
			0.200	0.185 ± 0.006	92.7	3.0
			2.000	1.932 ± 0.041	96.6	2.1

*Bile acids abbreviations are listed in table S1.

Table S5: Method validation parameters for bile acids.

Symbol	Bile acid*	Chemical formula	Exact mass [-H+]	RT [min]
S-GUDCA	Glycoursodeoxycholic acid sulfate	$C_{26}H_{43}NO_8S$	528.2637	2.45
S-GUDCA	Glycoursodeoxycholic acid sulfate	$C_{26}H_{43}NO_8S$	528.2637	2.72
G-CDCA	Chenodeoxycholic acid glucuronide	$C_{30}H_{48}O_{10}$	567.3175	2.60
G-CDCA	Chenodeoxycholic acid glucuronide	$C_{30}H_{48}O_{10}$	567.3175	2.82
G-CDCA	Chenodeoxycholic acid glucuronide	$C_{30}H_{48}O_{10}$	567.3175	3.57
G-CDCA	Chenodeoxycholic acid glucuronide	$C_{30}H_{48}O_{10}$	567.3175	4.55
S-TCDCA	Taurochenodeoxycholic acid sulfate	$C_{26}H_{45}NO_9S_2$	578.2463	2.62
G-TUDCA	Tauroursodeoxycholic acid glucuronide	$C_{32}H_{53}NO_{11}S$	658.3267	3.74
S-CDCA	Chenodeoxycholic acid sulfate	$C_{24}H_{40}O_7S$	471.2422	3.86
S-GLCA	Glycolithocholic acid sulfate	$C_{26}H_{43}NO_7S$	512.2688	4.19
KLCA	Ketolithocholic acid	$C_{24}H_{38}O_4$	389.2697	4.44
KLCA	Ketolithocholic acid	$C_{24}H_{38}O_4$	389.2697	5.04
KLCA	Ketolithocholic acid	$C_{24}H_{38}O_4$	389.2697	5.29
KLCA	Ketolithocholic acid	$C_{24}H_{38}O_4$	389.2697	6.07

*only one possible ID is shown.

Table S6: List of bile acids detected during untargeted screening of a plasma sample.

	Name*	Formula	Exact mass [-H$^+$]	Retention time (2-LPL) [min]	Retention time (1-LPL) [min]	Recovery ± SD [%]
	PC(12:0/0:0)	$C_{20}H_{42}NO_7P$	498.2838	6.62	7.00	99.2 ± 2.1
	PC(15:0/0:0)	$C_{23}H_{48}NO_7P$	540.3307	9.09	9.35	95.9 ± 4.3
	PC(17:1/0:0)	$C_{25}H_{50}NO_7P$	566.3464	9.39	9.63	93.3 ± 4.1
	PC(19:0/0:0)	$C_{27}H_{56}NO_7P$	596.3933	11.12	11.30	58.1 ± 9.2
	PC(20:0/0:0)	$C_{28}H_{58}NO_7P$	610.4090	11.50	11.66	48.3 ± 11.3
Soy LPC	PC(18:2/0:0)	$C_{26}H_{50}NO_7P$	578.3464	9.29	9.53	93.1 ± 2.7
	PC(16:0/0:0)	$C_{24}H_{50}NO_7P$	554.3464	9.70	9.94	92.9 ± 3.8
	PC(18:1/0:0)	$C_{26}H_{52}NO_7P$	580.3620	9.97	10.18	91.3 ± 4.4
	PC(18:0/0:0)	$C_{26}H_{54}NO_7P$	582.3777	10.71	10.90	71.1 ± 3.3
	PC(18:3/0:0)	$C_{26}H_{48}NO_7P$	576.3307	8.67	8.92	94.0 ± 2.5
Egg LPE	PE(18:0/0:0)	$C_{23}H_{48}NO_7P$	480.3096	10.75	10.92	60.7 ± 6.9
	PE(16:0/0:0)	$C_{21}H_{44}NO_7P$	452.2783	9.74	9.97	86.5 ± 2.6
	PE(18:1/0:0)	$C_{23}H_{46}NO_7P$	478.2939	10.00	10.22	85.5 ± 4.2
	PE(18:2/0:0)	$C_{23}H_{44}NO_7P$	476.2783	9.35	9.57	89.8 ± 2.0
	PE(16:1/0:0)	$C_{21}H_{42}NO_7P$	450.2626	8.87	9.10	91.7 ± 3.7
	PE(17:1/0:0)	$C_{22}H_{44}NO_7P$	464.2783	9.47	9.70	89.6 ± 4.4
Soy LPI	PI(16:0/0:0)	$C_{25}H_{49}O_{12}P$	571.2889	9.29	9.53	76.9 ± 4.6
	PI(18:0/0:0)	$C_{27}H_{53}O_{12}P$	599.3202	10.33	10.55	59.8 ± 3.2
	PI(18:2/0:0)	$C_{27}H_{49}O_{12}P$	595.2889	8.89	9.11	76.1 ± 4.9
	PI(18:1/0:0)	$C_{27}H_{51}O_{12}P$	597.3045	9.56	9.79	74.7 ± 6.6
	PI(20:4/0:0)	$C_{29}H_{49}O_{12}P$	619.2889	8.91	9.11	74.0 ± 4.9
	PS(16:0/0:0)	$C_{22}H_{44}NO_9P$	496.2681	9.30	9.56	78.2 ± 3.7
	PS(18:0/0:0)	$C_{24}H_{48}NO_9P$	524.2994	10.38	10.58	59.4 ± 4.2
	PS(18:1/0:0)	$C_{24}H_{46}NO_9P$	522.2838	9.62	9.84	77.0 ± 4.2
	PG(16:0/0:0)	$C_{22}H_{45}O_9P$	483.2729	9.48	9.70	86.4 ± 4.8
	PG(18:0/0:0)	$C_{24}H_{49}O_9P$	511.3042	10.48	10.67	71.1 ± 2.3
	PG(18:1/0:0)	$C_{24}H_{47}O_9P$	509.2885	9.73	9.93	82.8 ± 4.4
	PC (2:0/O-16:0)	$C_{26}H_{54}NO_7P$	582.3777	10.73	N/A	82.3 ± 4.4
	PC (2:0/O-18:0)	$C_{28}H_{58}NO_7P$	610.4090	11.54	N/A	62.7 ± 3.2
	PC (2:0/O-18:1)	$C_{28}H_{56}NO_7P$	608.3933	10.92	N/A	80.2 ± 2.6
	PC (O-16:0/0:0))	$C_{24}H_{52}NO_6P$	540.3671	10.46	N/A	87.4 ± 3.2
	PC (O-18:0/0:0))	$C_{26}H_{56}NO_6P$	568.3984	11.33	N/A	62.8 ± 5.2
Internal Standards	PC(13:0/0:0)	$C_{21}H_{44}NO_7P$	512.2994	7.57	7.90	100.8 ± 4.5
	PC(18:1-d$_7$/0:0)	$C_{26}H_{45}D_7NO_7P$	587.4059	9.94	10.16	93.0 ± 4.6
	PE(13:0/0:0)	$C_{18}H_{38}NO_7P$	410.2313	7.66	7.98	99.6 ± 2.4
	PE(18:1-d$_7$/0:0)	$C_{23}H_{39}D_7NO_7P$	485.3379	9.98	10.20	88.5 ± 4.2
	PI(13:0/0:0)	$C_{22}H_{43}O_{12}P$	529.2419	7.08	7.44	84.3 ± 4.7
	PI(17:1/0:0)	$C_{26}H_{49}O_{12}P$	583.2889	9.00	9.25	77.8 ± 5.9
	PS(13:0/0:0)	$C_{19}H_{38}NO_9P$	454.2212	7.14	7.48	88.7 ± 2.1
	PS(17:1/0:0)	$C_{23}H_{44}NO_9P$	508.2681	9.02	9.27	79.5 ± 5.5
	PG(13:0/0:0)	$C_{19}H_{39}O_9P$	441.2259	7.37	7.67	92.0 ± 2.8
	PG(17:1/0:0)	$C_{23}H_{45}O_9P$	495.2729	9.18	9.41	87.3 ± 4.5

* PC: Phosphatidylcholine; PE Phosphatidylethanol; PI; Phosphatidylinositiol; PS: Phosphatidylserine; PG: Phosphatidylglycerol. For each analyte, the number of carbon atoms and double bonds of the fatty acyl side chains are indicated in brackets using the format (sn-1/sn-2).

Table S7: List of lysophospholipid standards with their detection parameters.

Name*	RT [min]	Concentration [nmol/mL]	Interday CV [%]	Intraday CV [%]	Identity Confirmation
PC(12:0/0:0)	7.00	0.061 ± 0.002	3.2	2.1	Standard
PC(14:0/0:0)	8.68	3.217 ± 0.123	3.8	2.3	FA + PC fragments
PC(14:1/0:0)	7.60	0.027 ± 0.001	4.0	2.6	FA Fragment
PC(15:0/0:0)	9.35	0.894 ± 0.052	5.8	3.5	Standard
PC(0:0/16:0)	9.70	25.761 ± 1.612	6.3	4.7	Standard
PC(16:0/0:0)	9.94	147.748 ± 3.737	2.5	2.1	Standard
PC(16:1/0:0)	9.06	3.828 ± 0.150	3.9	2.2	Standard
PC(17:0/0:0)	10.45	1.084 ± 0.059	5.4	4.3	Standard
PC(17:1/0:0)	9.65	0.472 ± 0.016	3.4	2.7	Standard
PC(0:0/18:0)	10.71	4.872 ± 0.396	8.1	7.3	Standard
PC(18:0/0:0)	10.90	34.113 ± 2.381	7.0	5.1	Standard
PC(0:0/18:1)	9.97	3.769 ± 0.243	6.5	4.4	Standard
PC(18:1/0:0)	10.18	31.036 ± 0.569	1.8	1.6	Standard
PC(0:0/18:2)	9.29	8.221 ± 0.531	6.5	5.0	Standard
PC(18:2/0:0)	9.53	51.467 ± 2.266	4.4	3.3	Standard
PC(18:3/0:0)	8.92	0.654 ± 0.025	3.8	2.4	Standard
PC(19:0/0:0)	11.30	0.049 ± 0.006	12.6	8.3	Standard
PC(0:0/20:0)	11.50	0.008 ± 0.001	15.1	11.9	Standard
PC(20:0/0:0)	11.66	0.078 ± 0.010	12.7	8.1	Standard
PC(0:0/20:1)	10.87	0.025 ± 0.003	10.2	8.5	FA Fragment
PC(20:1/0:0)	11.05	0.219 ± 0.015	6.9	5.3	FA + PC fragments
PC(20:2/0:0)	10.52	0.329 ± 0.010	3.2	2.9	FA Fragment
PC(20:3/0:0)_01	9.97	4.938 ± 0.264	5.3	2.7	FA + PC fragments
PC(20:3/0:0)_02	10.14	0.459 ± 0.015	3.3	3.0	FA + PC fragments
PC(0:0/20:4)	9.31	3.293 ± 0.253	7.7	5.8	FA + PC fragments
PC(20:4/0:0)	9.52	21.535 ± 1.204	5.6	3.8	FA + PC fragments
PC(20:5/0:0)	8.91	1.192 ± 0.047	4.0	2.4	FA + PC fragments
PC(22:0/0:0)	12.28	0.014 ± 0.002	17.1	11.2	FA Fragment
PC(22:1/0:0)	11.78	0.009 ± 0.002	16.2	11.8	No confirmation
PC(22:4/0:0)	10.37	0.304 ± 0.012	3.8	2.4	FA + PC fragments
PC(22:5/0:0)_01	9.87	0.832 ± 0.047	5.7	3.4	FA + PC fragments
PC(22:5/0:0)_02	10.06	0.278 ± 0.013	4.8	3.0	FA + PC fragments

PC(22:6/0:0)	9.53	2.753 ± 0.1721	6.3	3.7	FA + PC fragments
PC (O-16:0/0:0)	10.46	0.835 ± 0.034	4.1	3.1	Standard
PC (O-16:1/0:0)	10.39	0.931 ± 0.034	3.7	3.0	PC fragments
PC (O-18:0/0:0)	11.33	0.134 ± 0.014	10.3	7.5	Standard
PC (O-18:1/0:0)	10.66	0.678 ± 0.020	3.0	2.9	PC fragments
PE(12:0/0:0)	7.08	0.003 ± 0.001	7.5	6.3	FA Fragment
PE(14:0/0:0)	8.74	0.026 ± 0.001	4.8	2.8	FA Fragment
PE(0:0/16:0)	9.74	0.200 ± 0.025	12.4	6.4	Standard
PE(16:0/0:0)	9.97	3.879 ± 0.216	5.6	4.5	Standard
PE(16:1/0:0)	9.10	0.107 ± 0.005	4.5	3.2	Standard
PE(17:1/0:0)	9.70	0.024 ± 0.002	9.7	6.1	Standard
PE(0:0/18:0)	10.75	0.208 ± 0.022	10.4	6.1	Standard
PE(18:0/0:0)	10.92	2.356 ± 0.318	13.5	8.8	Standard
PE(0:0/18:1)	10.00	0.426 ± 0.039	9.2	6.8	Standard
PE(18:1/0:0)	10.22	2.499 ± 0.088	3.5	1.7	Standard
PE(0:0/18:2)	9.35	0.518 ± 0.073	14.0	7.3	Standard
PE(18:2/0:0)	9.57	5.811 ± 0.476	8.2	6.5	Standard
PE(18:3/0:0)	8.99	0.091 ± 0.004	4.4	2.8	FA + PE fragments
PE(20:1/0:0)	11.08	0.010 ± 0.001	12.5	9.0	PE fragments
PE(20:2/0:0)	10.59	0.019 ± 0.003	13.5	9.8	FA Fragment
PE(20:3/0:0)_01	10.00	0.424 ± 0.027	6.4	6.3	FA + PE fragments
PE(20:3/0:0)_02	10.19	0.041 ± 0.001	2.8	2.9	FA + PE fragments
PE(0:0/20:4)	9.36	0.425 ± 0.061	14.4	7.2	FA + PE fragments
PE(20:4/0:0)	9.55	6.219 ± 0.487	7.8	6.8	FA + PE fragments
PE(20:5/0:0)	8.98	0.172 ± 0.007	3.9	2.5	FA + PE fragments
PE(22:4/0:0)	10.43	0.069 ± 0.008	11.6	6.8	FA + PE fragments
PE(22:5/0:0)_01	9.94	1.155 ± 0.075	6.5	5.3	FA + PE fragments
PE(22:5/0:0)_02	10.15	0.157 ± 0.014	8.8	4.4	FA + PE fragments
PE(22:6/0:0)	9.58	1.646 ± 0.124	7.5	6.4	FA + PE fragments
PG(16:0/0:0)	9.70	0.026 ± 0.003	12.9	8.3	Standard
PG(16:1/0:0)	8.84	0.003 ± 0.001	8.5	6.8	FA Fragment
PG(18:0/0:0)	10.67	0.017 ± 0.003	18.6	12.7	Standard
PG(18:1/0:0)	9.93	0.153 ± 0.012	7.8	4.7	Standard
PG(18:2/0:0)	9.30	0.025 ± 0.002	6.2	4.2	FA + PG fragments

PG(20:3/0:0)	9.73	0.002 ± 0.001	21.0	11.4	FA + PG fragments
PG(20:4/0:0)	9.29	0.011 ± 0.001	6.3	4.8	FA + PG fragments
PG(20:5/0:0)	8.70	0.001 ± 0.001	27.0	25.9	No confirmation
PG(22:6/0:0)	9.31	0.004 ± 0.001	8.9	5.6	PG fragments
PI(14:0/0:0)	8.25	0.003 ± 0.001	9.1	5.9	No confirmation
PI(16:0/0:0)	9.53	0.064 ± 0.007	10.2	7.4	Standard
PI(16:1/0:0)	8.64	0.041 ± 0.002	5.9	3.6	FA Fragment
PI(18:0/0:0)	10.55	0.181 ± 0.036	19.7	11.0	Standard
PI(0:0/18:1)	9.56	0.037 ± 0.004	10.2	6.0	Standard
PI(18:1/0:0)	9.79	0.147 ± 0.007	4.6	3.6	Standard
PI(0:0/18:2)	8.89	0.025 ± 0.001	4.8	3.4	Standard
PI(18:2/0:0)	9.11	0.207 ± 0.009	4.5	2.4	Standard
PI(18:3/0:0)	8.51	0.003 ± 0.001	10.1	7.1	No confirmation
PI(20:3/0:0)_01	9.57	0.125 ± 0.014	11.1	5.9	FA + PI fragments
PI(20:3/0:0)_02	9.76	0.019 ± 0.002	8.4	4.2	FA + PI fragments
PI(0:0/20:4)	8.91	0.065 ± 0.003	5.4	4.0	Standard
PI(20:4/0:0)	9.11	0.531 ± 0.031	5.9	3.0	Standard
PI(20:5/0:0)	8.51	0.003 ± 0.001	9.6	6.1	No confirmation
PI(22:5/0:0)_01	9.50	0.041 ± 0.003	8.5	6.9	No confirmation
PI(22:5/0:0)_02	9.71	0.005 ± 0.001	6.4	5.1	No confirmation
PI(22:6/0:0)	9.15	0.020 ± 0.001	4.3	2.7	PI Fragment
PS(18:0/0:0)	10.58	0.006 ± 0.001	22.6	15.8	Standard
PS(18:1/0:0)	9.84	0.003 ± 0.001	12.0	11.4	Standard
PS(18:2/0:0)	9.17	0.002 ± 0.001	13.4	12.3	No confirmation
PS(20:4/0:0)	9.15	0.016 ± 0.001	4.5	3.7	No confirmation
PS(22:6/0:0)	9.20	0.007 ± 0.001	5.5	5.1	No confirmation

* PC: Phosphatidylcholine; PE Phosphatidylethanol; PI; Phosphatidylinositiol; PS: Phosphatidylserine; PG: Phosphatidylglycerol; LPL: lysophospholipid; FA: fatty acid. For each analyte, the number of carbon atoms and double bonds of the fatty acyl side chains are indicated in brackets using the format (sn-1/sn-2).

Table S8: List of lysophospholipid detected in plasma during validation experiment.

Lipid Name*	Contrast	Difference	SE	df	t.ratio	p.value
PC(22:4/0:0)	CID1,non-responders - CID2,non-responders	0.06612	0.03442152	196	1.92089131	0.39254086

PC(22:4/0:0)	CID 1,non-responders - CID 3,non-responders	0.00228	0.03442152	196	0.06623763	0.99999982
PC(22:4/0:0)	CID 1,non-responders - CID 1,responders	-0.1410539	0.04055275	245.493446	-3.4782809	0.00777836
PC(22:4/0:0)	CID 1,non-responders - CID 2,responders	0.02848613	0.04055275	245.493446	0.70244627	0.98152209
PC(22:4/0:0)	CID 1,non-responders - CID 3,responders	-0.0212539	0.04055275	245.493446	-0.5241042	0.99518089
PC(22:4/0:0)	CID 2,non-responders - CID 3,non-responders	-0.06384	0.03442152	196	-1.8546537	0.43338107
PC(22:4/0:0)	CID 2,non-responders - CID 1,responders	-0.2071739	0.04055275	245.493446	-5.1087497	9.62E-06
PC(22:4/0:0)	CID 2,non-responders - CID 2,responders	-0.0376339	0.04055275	245.493446	-0.9280225	0.93898416
PC(22:4/0:0)	CID 2,non-responders - CID 3,responders	-0.0873739	0.04055275	245.493446	-2.154573	0.2631549
PC(22:4/0:0)	CID 3,non-responders - CID 1,responders	-0.1433339	0.04055275	245.493446	-3.534504	0.00642088
PC(22:4/0:0)	CID 3,non-responders - CID 2,responders	0.02620613	0.04055275	245.493446	0.64622321	0.98730811
PC(22:4/0:0)	CID 3,non-responders - CID 3,responders	-0.0235339	0.04055275	245.493446	-0.5803273	0.99224986
PC(22:4/0:0)	CID 1,responders - CID 2,responders	0.16954	0.03442152	196	4.92540702	2.62E-05
PC(22:4/0:0)	CID 1,responders - CID 3,responders	0.1198	0.03442152	196	3.4803808	0.00800791
PC(22:4/0:0)	CID 2,responders - CID 3,responders	-0.04974	0.03442152	196	-1.4450262	0.69941936

PE(17:1/0:0)	CID 1,non-responders - CID 2,non-responders	0.00112	3.77E-04	196	2.97033549	0.03871112
PE(17:1/0:0)	CID 1,non-responders - CID 3,non-responders	-1.40E-04	3.77E-04	196	-0.3712919	0.99906867
PE(17:1/0:0)	CID 1,non-responders - CID 1,responders	-0.001617	4.86E-04	220.850097	-3.3290267	0.01291767
PE(17:1/0:0)	CID 1,non-responders - CID 2,responders	0.00194301	4.86E-04	220.850097	4.00020714	0.00120208
PE(17:1/0:0)	CID 1,non-responders - CID 3,responders	-2.17E-04	4.86E-04	220.850097	-0.4467437	0.99773559
PE(17:1/0:0)	CID 2,non-responders - CID 3,non-responders	-0.00126	3.77E-04	196	-3.3416274	0.01261576
PE(17:1/0:0)	CID 2,non-responders - CID 1,responders	-0.002737	4.86E-04	220.850097	-5.6348531	7.90E-07
PE(17:1/0:0)	CID 2,non-responders - CID 2,responders	8.23E-04	4.86E-04	220.850097	1.69438077	0.53696215
PE(17:1/0:0)	CID 2,non-responders - CID 3,responders	-0.001337	4.86E-04	220.850097	-2.7525701	0.06928073
PE(17:1/0:0)	CID 3,non-responders - CID 1,responders	-0.001477	4.86E-04	220.850097	-3.0407984	0.03128398
PE(17:1/0:0)	CID 3,non-responders - CID 2,responders	0.00208301	4.86E-04	220.850097	4.28843544	3.83E-04
PE(17:1/0:0)	CID 3,non-responders - CID 3,responders	-7.70E-05	4.86E-04	220.850097	-0.1585154	0.99998595
PE(17:1/0:0)	CID 1,responders - CID 2,responders	0.00356	3.77E-04	196	9.44142353	1.33E-13
PE(17:1/0:0)	CID 1,responders - CID 3,responders	0.0014	3.77E-04	196	3.71291937	0.00358904

PE(17:1/0:0)	CID 2,responders - CID 3,responders	-0.00216	3.77E-04	196	-5.7285042	5.61E-07
PC(22:5/0:0)_01	CID 1,non-responders - CID 2,non-responders	0.2321	0.11651915	196	1.99194721	0.35067191
PC(22:5/0:0)_01	CID 1,non-responders - CID 3,non-responders	-0.02538	0.11651915	196	-0.2178183	0.99993189
PC(22:5/0:0)_01	CID 1,non-responders - CID 1,responders	-0.3754804	0.14486175	226.370877	-2.5919917	0.10343326
PC(22:5/0:0)_01	CID 1,non-responders - CID 2,responders	0.11731955	0.14486175	226.370877	0.80987256	0.96550782
PC(22:5/0:0)_01	CID 1,non-responders - CID 3,responders	-0.1292404	0.14486175	226.370877	-0.8921641	0.94809412
PC(22:5/0:0)_01	CID 2,non-responders - CID 3,non-responders	-0.25748	0.11651915	196	-2.2097655	0.23797368
PC(22:5/0:0)_01	CID 2,non-responders - CID 1,responders	-0.6075804	0.14486175	226.370877	-4.1942091	5.57E-04
PC(22:5/0:0)_01	CID 2,non-responders - CID 2,responders	-0.1147804	0.14486175	226.370877	-0.7923448	0.96860959
PC(22:5/0:0)_01	CID 2,non-responders - CID 3,responders	-0.3613404	0.14486175	226.370877	-2.4943814	0.13010146
PC(22:5/0:0)_01	CID 3,non-responders - CID 1,responders	-0.3501004	0.14486175	226.370877	-2.4167902	0.15482885
PC(22:5/0:0)_01	CID 3,non-responders - CID 2,responders	0.14269955	0.14486175	226.370877	0.9850741	0.92234118
PC(22:5/0:0)_01	CID 3,non-responders - CID 3,responders	-0.1038604	0.14486175	226.370877	-0.7169626	0.9797441
PC(22:5/0:0)_01	CID 1,responders - CID 2,responders	0.4928	0.11651915	196	4.22934762	5.09E-04

PC(22:5/0:0)_01	CID 1,responders - CID 3,responders	0.24624	0.11651915	196	2.11330065	0.28474022
PC(22:5/0:0)_01	CID 2,responders - CID 3,responders	-0.24656	0.11651915	196	-2.116047	0.28333723

* PC: Phosphatidylcholine; PE Phosphatidylethanol; PI; Phosphatidylinositiol; PS: Phosphatidylserine; PG: Phosphatidylglycerol. For each analyte, the number of carbon atoms and double bonds of the fatty acyl side chains are indicated in brackets using the format (sn-1/sn-2).

Table S9: Linear mixed effect model, post-hoc analyses (Tukey's HSD). CID: clinical intervention day. CID1: baseline, CID2: After 8-week low-caloric diet (LCD) intervention, CID3: After 6-month weight maintenance phase

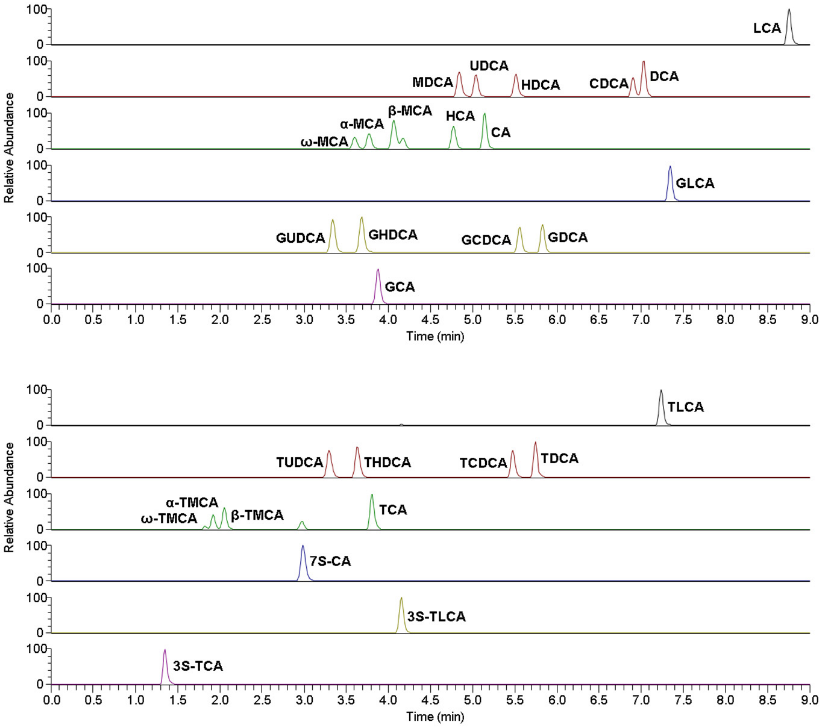

Figure S1: Extracted chromatograms of bile acids spiked in a plasma sample. For bile acids abbreviations, please refer to table S1

PC(12:0/0:0)	PC(14:0/0:0)
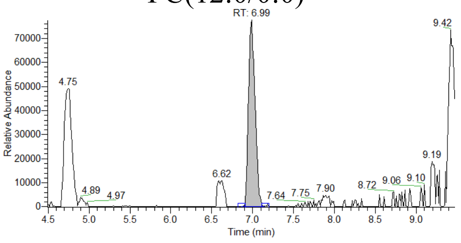	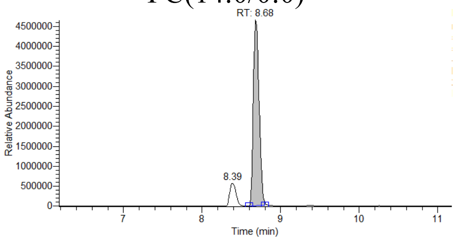

PC(14:1/0:0)

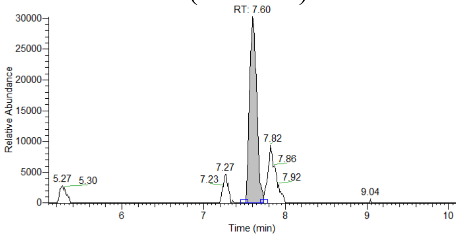

PC(15:0/0:0)

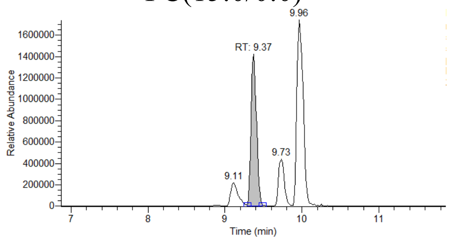

PC(0:0/16:0)

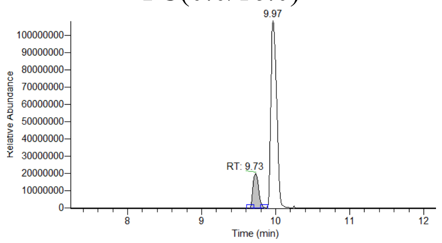

PC(16:0/0:0)

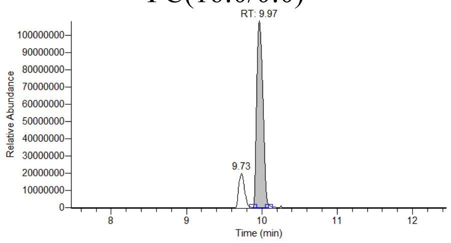

PC(16:1/0:0)

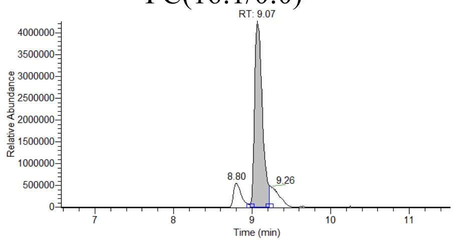

PC(17:0/0:0)

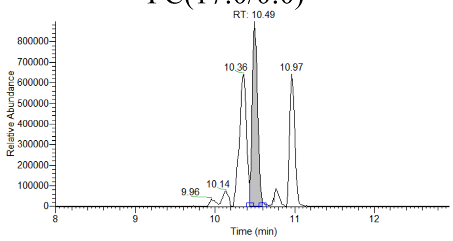

PC(17:1/0:0)

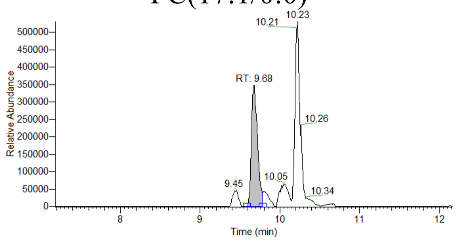

PC(0:0/18:0)

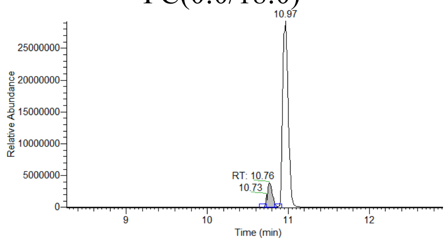

PC(18:0/0:0)

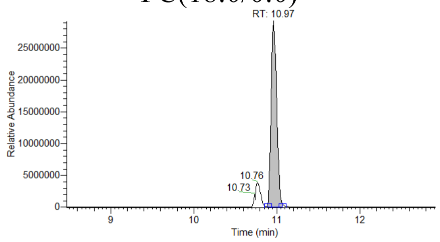

PC(0:0/18:1)

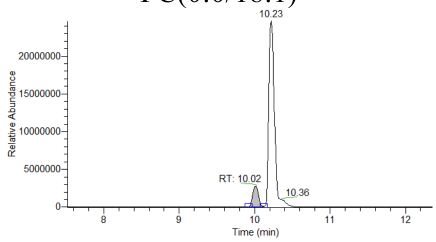

PC(18:1/0:0)

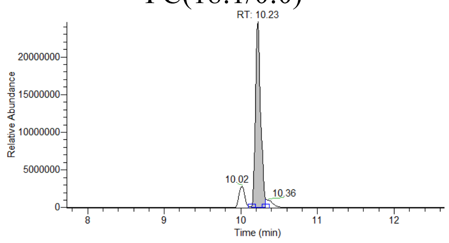

PC(0:0/18:2)

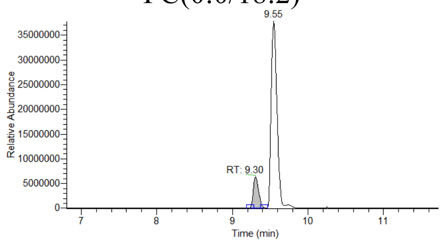

PC(18:2/0:0)

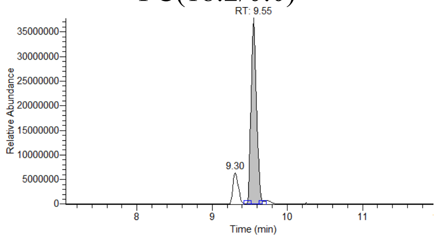

PC(18:3/0:0)

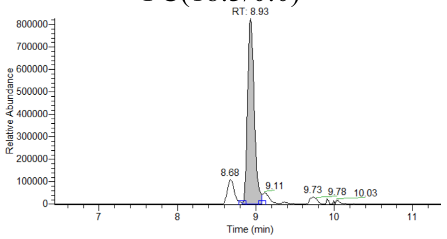

PC(19:0/0:0)

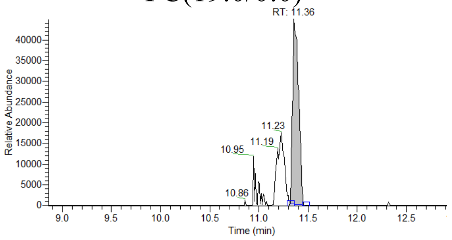

PC(0:0/20:0)

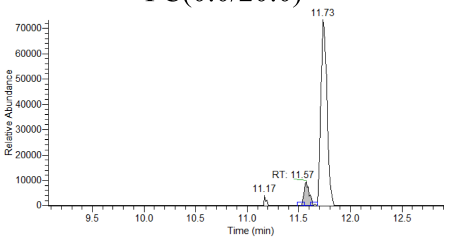

PC(20:0/0:0)

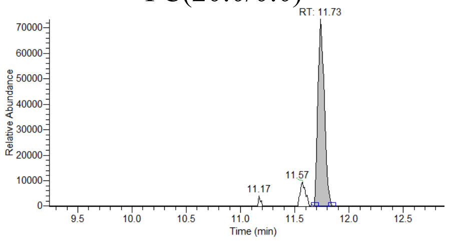

PC(0:0/20:1)

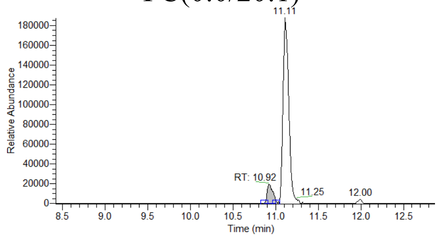

PC(20:1/0:0)

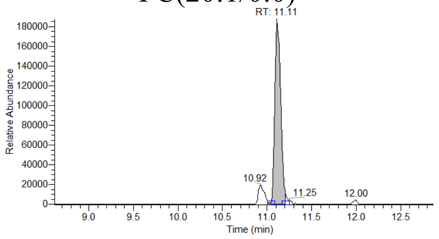

PC(20:2/0:0)

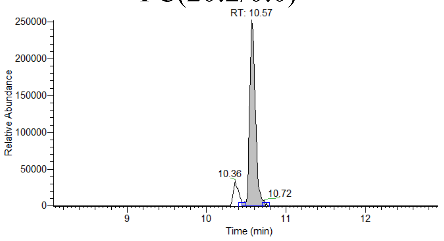

PC(20:3/0:0)_01

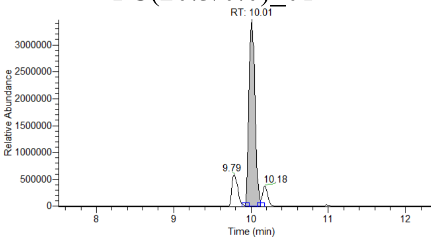

PC(20:3/0:0)_02

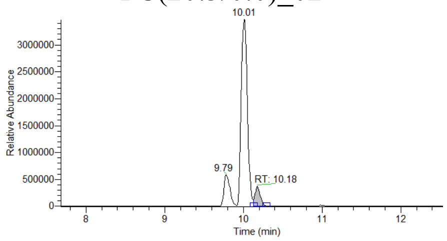

PC(0:0/20:4)

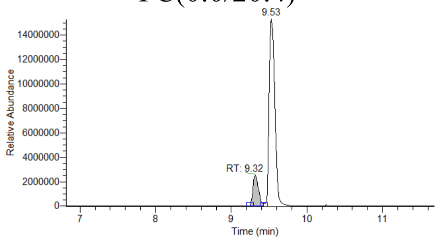

PC(20:4/0:0)

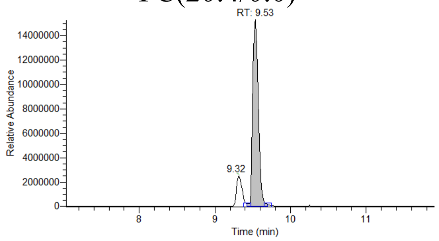

PC(20:5/0:0)

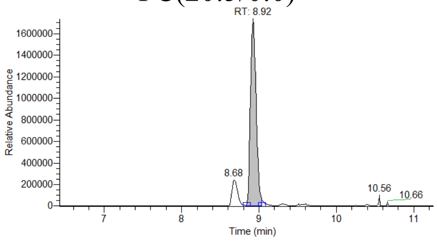

PC(22:0/0:0)

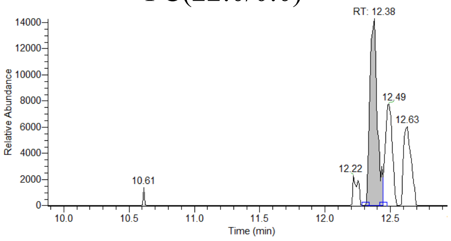

PC(22:1/0:0)

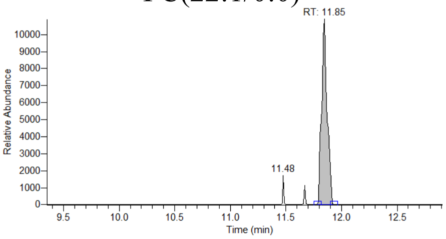

PC(22:4/0:0)

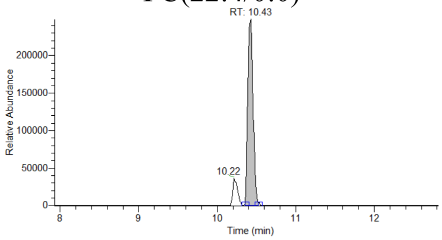

PC(22:5/0:0)_01

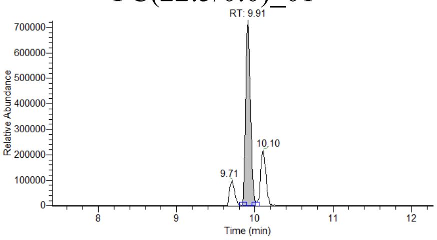

PC(22:5/0:0)_02

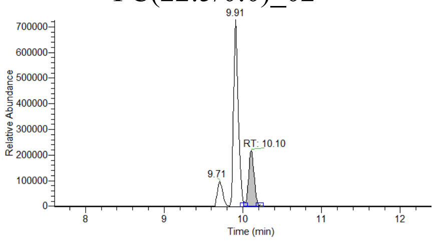

PC(22:6/0:0)

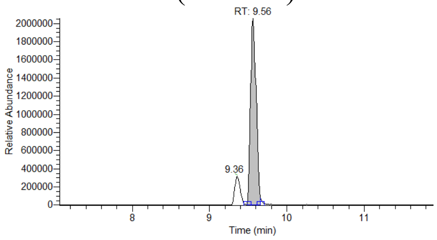

PC (O-16:0/0:0)

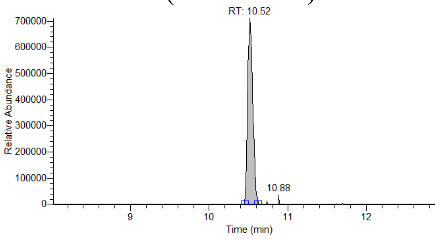

PC (O-16:1/0:0)

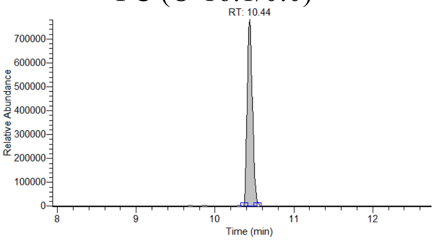

PC (O-18:0/0:0)

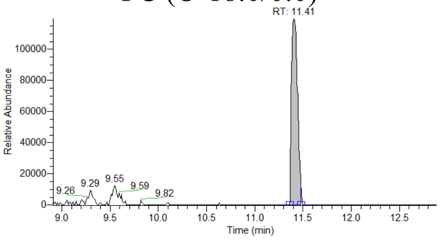

PC (O-18:1/0:0)

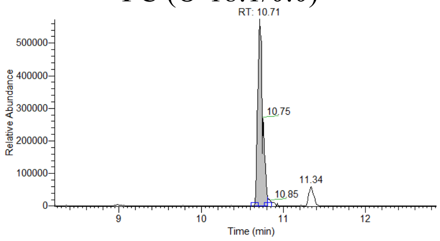

PE(12:0/0:0)

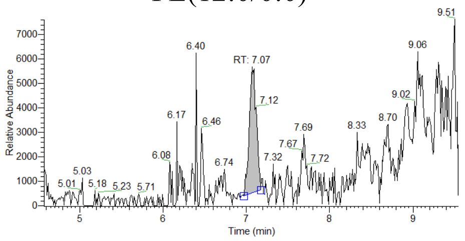

PE(14:0/0:0)

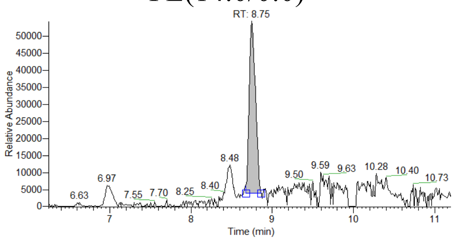

PE(0:0/16:0)

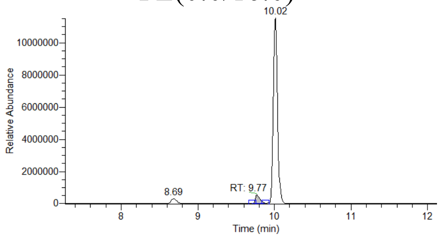

PE(16:0/0:0)

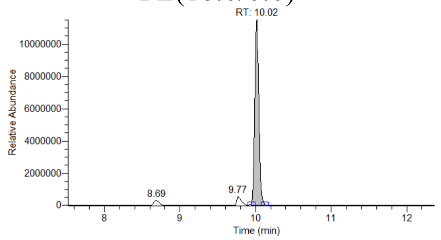

PE(16:1/0:0)

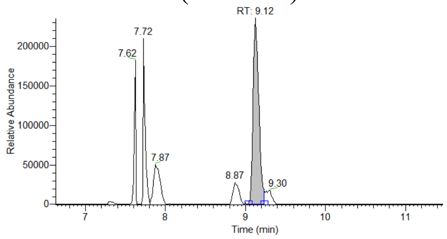

PE(17:1/0:0)

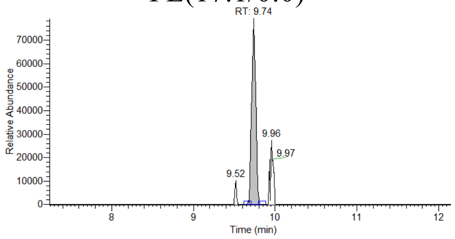

PE(0:0/18:0)

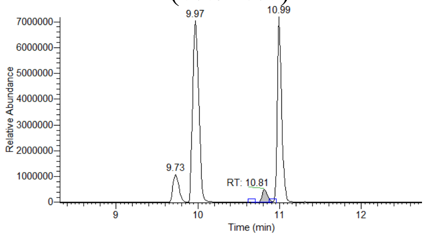

PE(18:0/0:0)

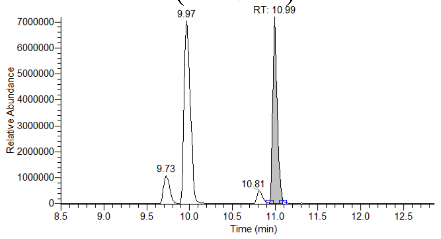

PE(0:0/18:1)

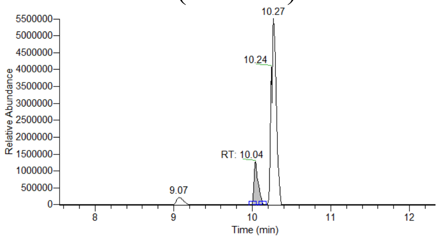

PE(18:1/0:0)

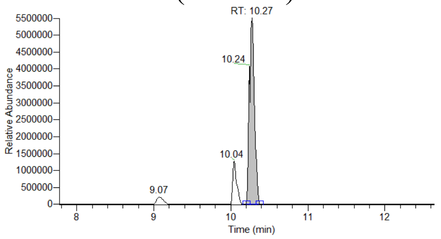

PE(0:0/18:2)

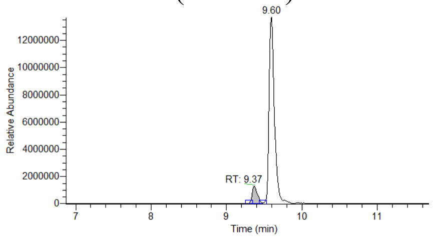

PE(18:2/0:0)

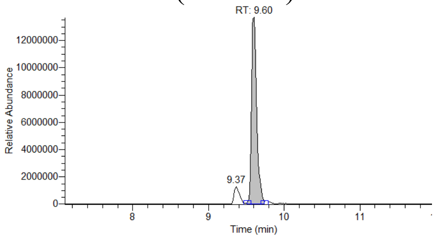

PE(18:3/0:0)

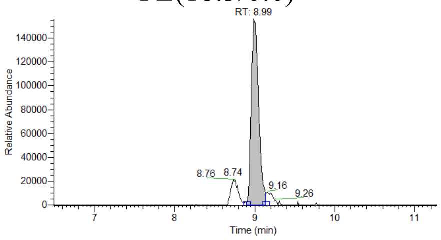

PE(20:1/0:0)

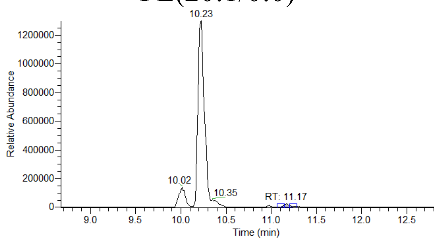

PE(20:2/0:0)

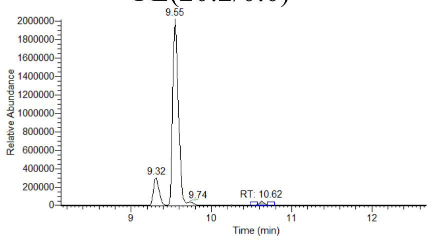

PE(20:3/0:0)_01

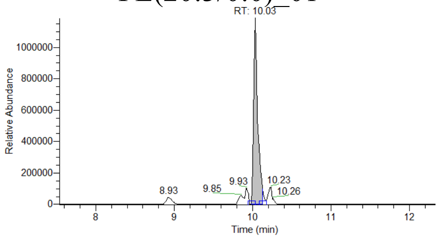

PE(20:3/0:0)_02

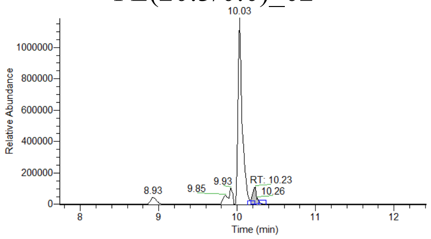

PE(0:0/20:4)

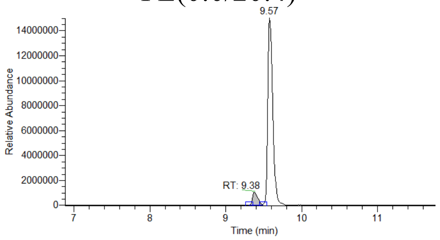

PE(20:4/0:0)

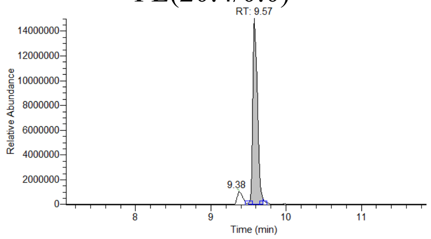

PE(20:5/0:0)

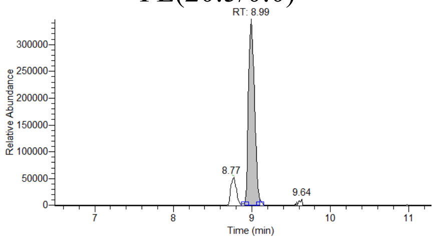

PE(22:4/0:0)

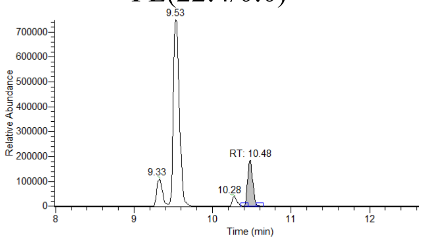

PE(22:5/0:0)_01

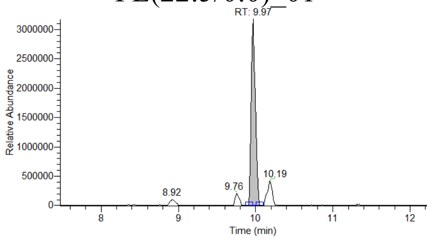

PE(22:5/0:0)_02

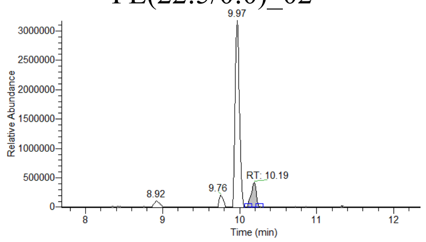

PE(22:6/0:0)

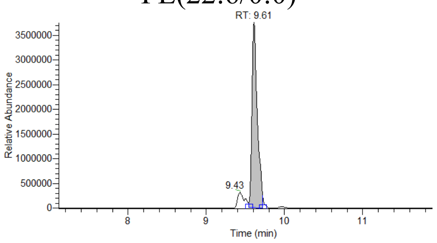

PG(16:0/0:0)

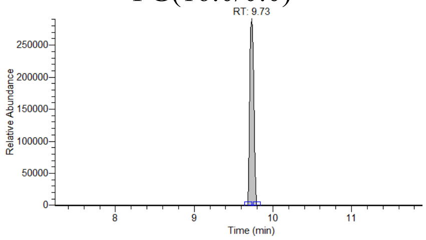

PG(16:1/0:0)

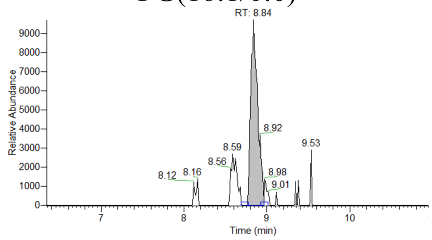

PG(18:0/0:0)

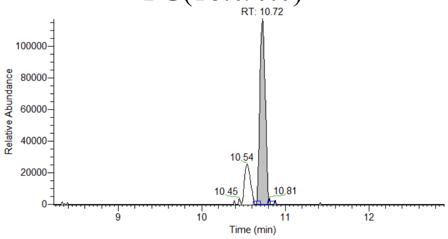

PG(18:1/0:0)

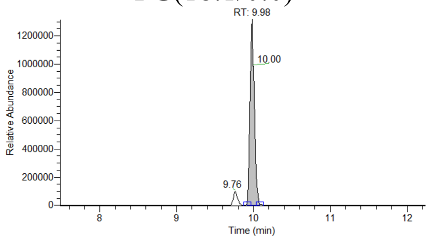

PG(18:2/0:0)

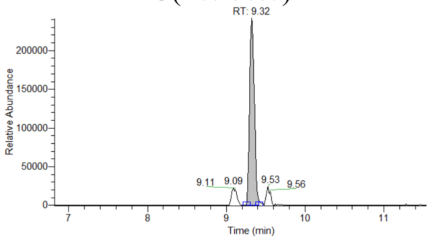

PG(20:3/0:0)

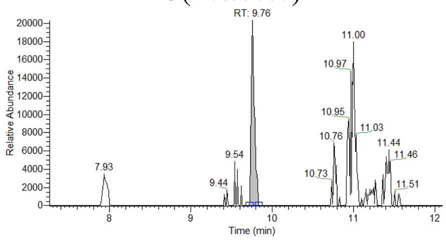

PG(20:4/0:0)

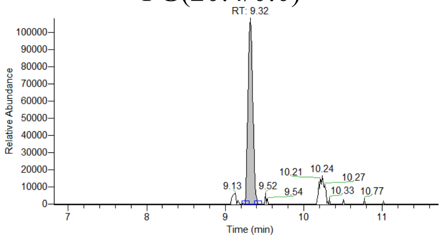

PG(20:5/0:0)

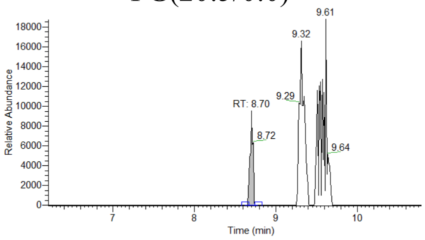

PG(22:6/0:0)

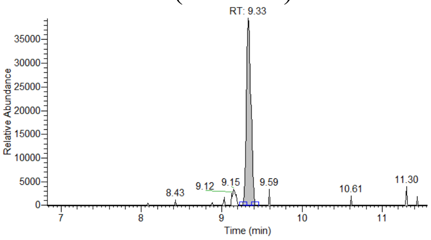

PI(14:0/0:0)

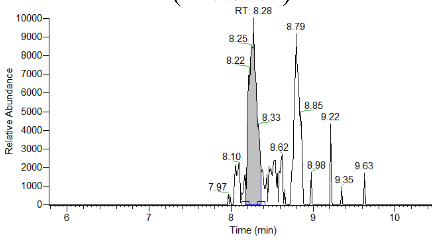

PI(16:0/0:0)

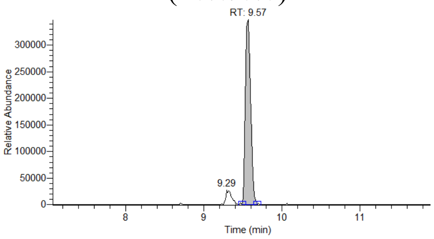

PI(16:1/0:0)

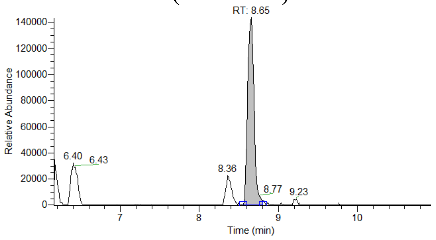

PI(18:0/0:0)

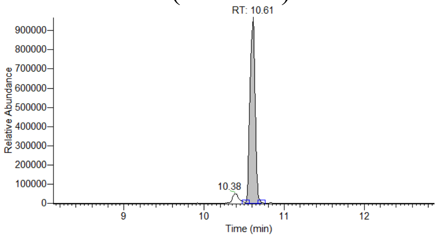

PI(0:0/18:1)

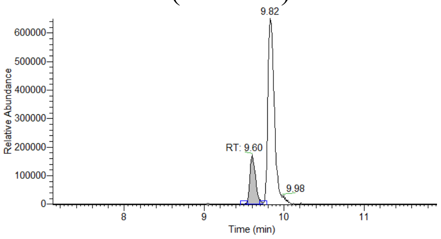

PI(18:1/0:0)

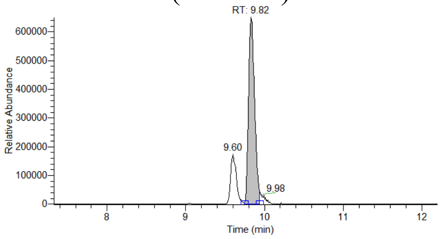

PI(0:0/18:2)

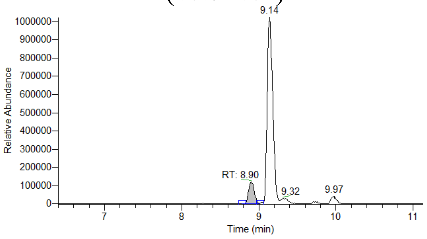

PI(18:2/0:0)

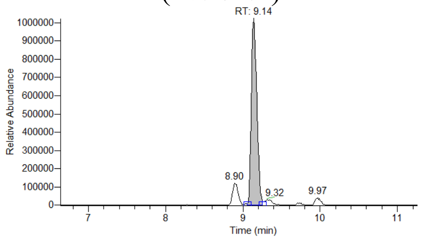

PI(18:3/0:0)

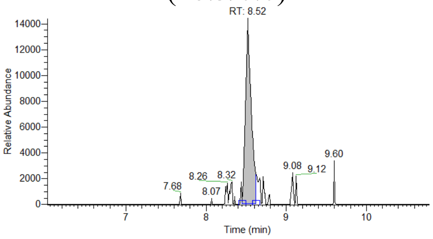

PI(20:3/0:0)_01

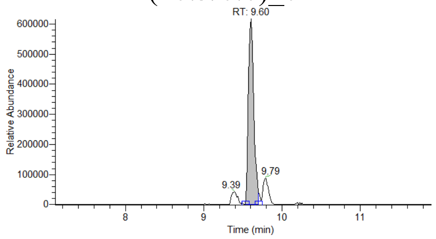

PI(20:3/0:0)_02

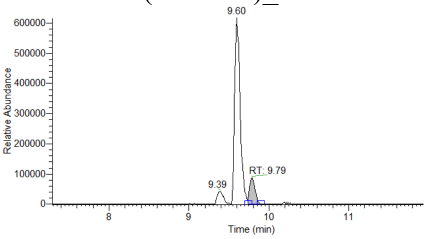

PI(0:0/20:4)

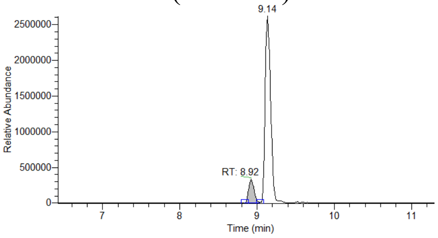

PI(20:4/0:0)

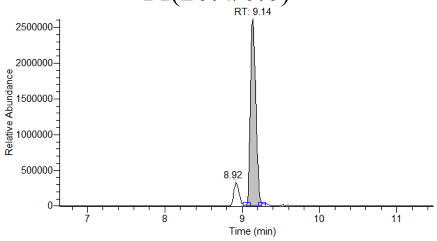

PI(20:5/0:0)

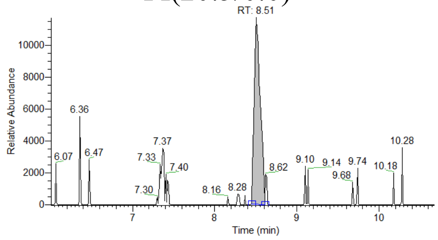

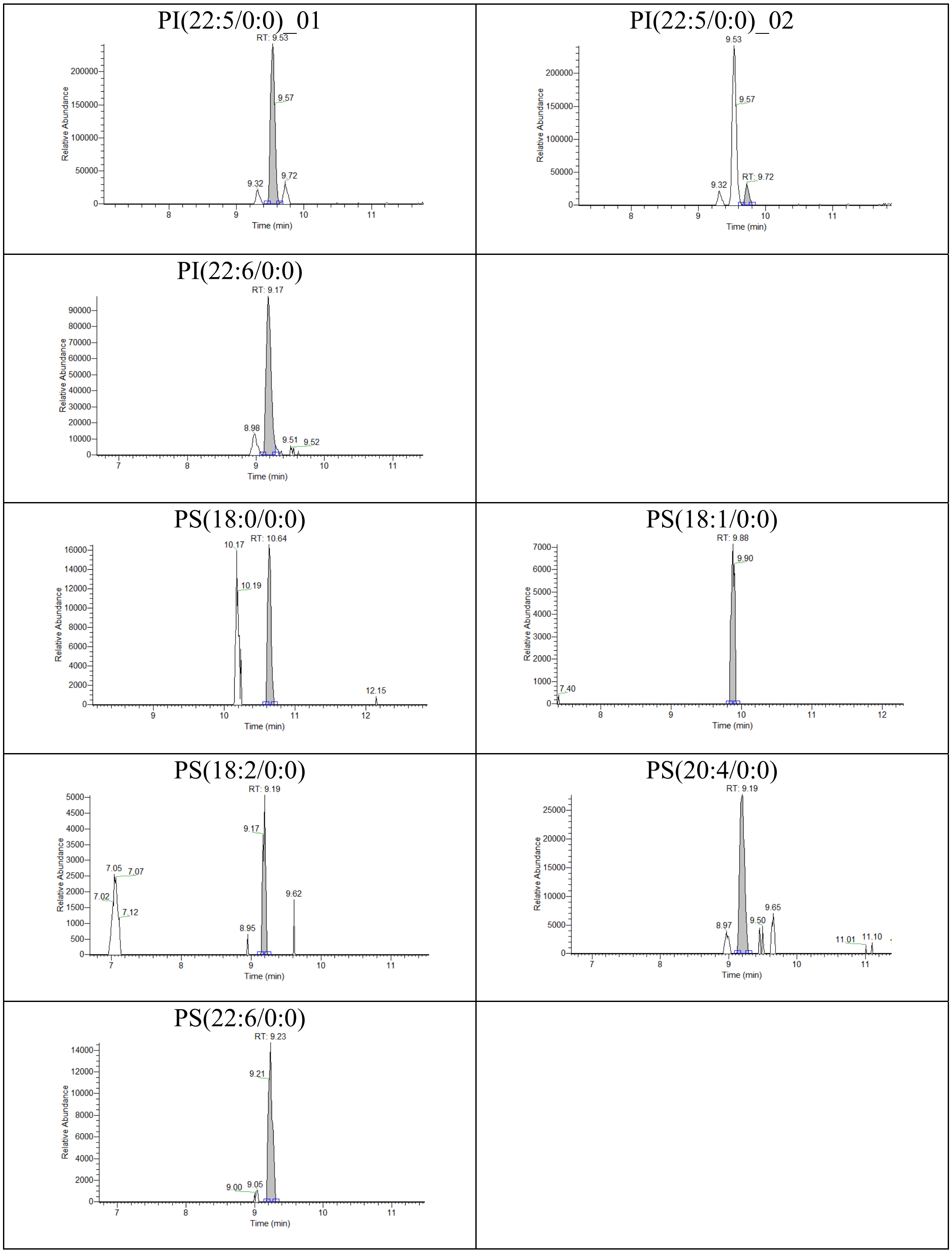

Figure S2: Extracted chromatograms of lysophospholipids in a plasma sample.

PC: Phosphatidylcholine; PE Phosphatidylethanol; PI; Phosphatidylinositiol; PS: Phosphatidylserine; PG: Phosphatidylglycerol. For each analyte, the number of carbon atoms and double bonds of the fatty acyl side chains are indicated in brackets using the format (sn-1/sn-2).
